# *Sinocurculigo*, a New Genus of Hypoxidaceae from China Based on Molecular and Morphological Evidence

**DOI:** 10.1371/journal.pone.0038880

**Published:** 2012-06-27

**Authors:** Ke-Wei Liu, Gao-Chang Xie, Li-Jun Chen, Xin-Ju Xiao, Yu-Yun Zheng, Jing Cai, Jun-Wen Zhai, Guo-Qiang Zhang, Zhong-Jian Liu

**Affiliations:** 1 Shenzhen Key Laboratory for Orchid Conservation and Utilization, The National Orchid Conservation Center of China and The Orchid Conservation and Research Center of Shenzhen, Shenzhen, China; 2 The Center for Biotechnology and BioMedicine, Graduate School at Shenzhen, Tsinghua University, Shenzhen, China; 3 South China Botanical Garden, Chinese Academy of Sciences, Guangzhou, China; 4 College of Forestry, South China Agricultural University, Guangzhou, China; University College London, United Kingdom

## Abstract

**Background:**

The monocot family Hypoxidaceae consists of nine genera with nearly 200 species. They occur mostly in the Southern Hemisphere with only a few species in the Northern Hemisphere, of which three genera, *Hypoxis*, *Molineria*, and *Curculigo*, with eight species are distributed in China. Recently, we have found a hypoxid-like plant in China that is quite different in floral structure from any of the three genera and even of the known taxa in Hypoxidaceae.

**Methodology/Principal Findings:**

In addition to morphological analysis, we performed maximum parsimony, maximum likelihood, and Bayesian inference analyses based on fragments of the chloroplast *matK* and *rbcL* genes of 60 taxa in 12 families representing all major clades of the Hypoxidaceae alliance. Results showed that Hypoxidaceae is monophyletic and and that the new plant belongs to it, forming a distinct clade within the family Hypoxidaceae as a sister of *Molineria*. Phylogeny of the Hypoxidaceae family was constructed based on a combined matrix of the chloroplast *rbcL*, *trnS*-*G*, and *trnL-F* regions of 59 taxa in Hypoxidaceae and its alliance. Findings of the molecular investigation is consistent with those of the morphological analysis.

**Conclusions/Significance:**

Based on the results of our molecular and morphological analyses in the present study, we propose a new genus, *Sinocurculigo*.

## Introduction

The economic importance of the monocot family Hypoxidaceae lies mostly in their use as traditional medicines. Some species of this family could be beneficial in the treatment of diseases, including HIV and certain tumors [1]–[5]. It occurs mostly in the Southern Hemisphere with only a few species in the Northern Hemisphere. The family consists of nine genera with nearly 200 species [6]–[9], of which three genera, *Hypoxis*, *Curculigo*, and *Molineria*, and eight species occur in China [8], [9]. General flower structure of the family is characterized by six perianthial segments in two wheels, six stamens, and a trimerous gynoecium [8], [10]. The classification of Hypoxidaceae has been variously treated by different authors based on morphological and DNA data. Fox example, Thompson [11] separated the southern African genera, placing *Hypoxis* and *Rhodohypoxis* in one group and *Empodium*, *Spiloxene*, and *Pauridia* in another. Rudall et al. [6] confirmed that Hypoxidaceae is monophyly composed of nine genera by cladistic analyses of *rbcL* DNA sequences. Nordal [12], based on geographical and basic morphological information, suggested two primary groupings of the family: one centering around the Indian Ocean with genera *Curculigo*, *Hypoxidia*, and *Molineria*; another occurring mainly in southern Africa with *Empodium*, *Pauridia*, *Rhodohypoxis*, *Saniella*, *Spiloxene*, and *Hypoxis*. *Hypoxis* was regarded as sister to the southern African genera because of its much wider distribution. In 2011, Kocyan et al. [7] reconstructed the phylogenetic relationships of the family by using four plastid DNA regions and identified three well-supported major clades: the *Curculigo*, *Hypoxis* and *Pauridia-Empodium* clades. These clades comprise a complex assemblage of the genera in the family. Recently, we have discovered a new member of the family in southern China that is quite different in floral features from any of the known taxa in Hypoxidaceae [13]. Clarification requires both molecular and morphological analyses and consideration of phytogeography.

**Figure 1:**
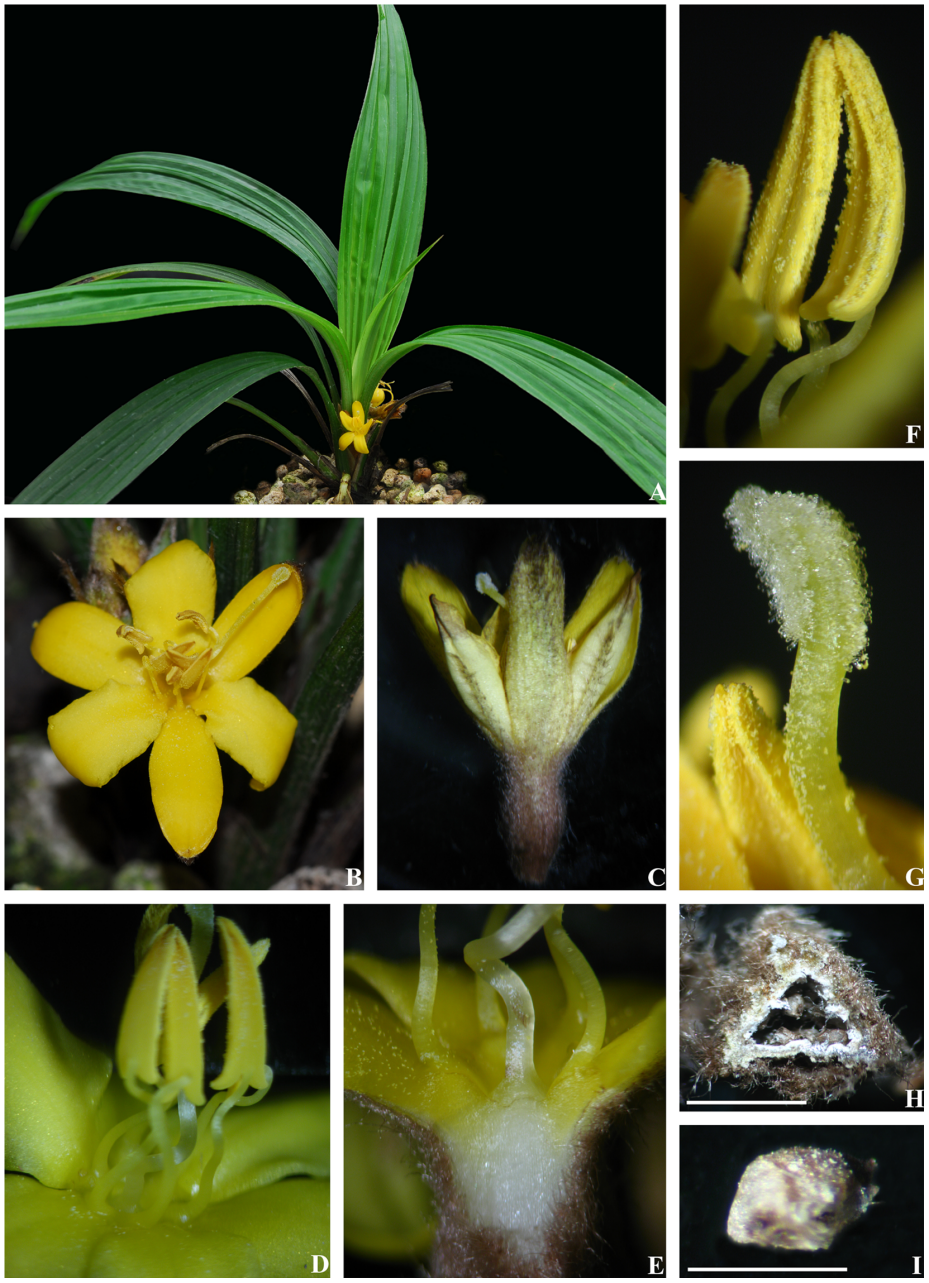
*Sinocurculigo taishanica*. A. Flowering plant; **B.** Flower; **C.** Flower, side view; D. Stamens, side view; **E.** Base of style, stamens, sepals and petals, longitudinal section of a flower. **F.** Anthers, front and side view; **G.** Stigma, side view; **H.** Ovary, transverse section, bar = 3 mm; **I.** Seed, bar = 0.5 mm.

## Results

### Morphological Analysis

The new hypoxid entity was collected from Taishan in southern Guangdong province, China (Figure S1). At the beginning of our discovery of this hypoxid, a minute comparison between our hypoxid plant and many members of Hypoxidaceae revealed that the new plant is characterized by sepals distinguishable from petals and long filaments arising from style base and adnate to the perianth segments with their basal parts (1–1.5 mm long). It has an obscure 3-ridged style with two rows of glandular hairs along the uppermost part of each obscure ridge. These hairs then come together at the top of the style, forming a terminal stigma composed of three inverted U-shaped hairy piles. The plant had unilocular ovary with three parietal placentas and fruit contains numerous seeds which are papillate on the surface (Figure 1). These features distinguish this taxon from all hypoxid genera currently known to us.

The new plant is no doubt a very remarkable hypoxid that is difficult to place in any known genera of Hypoxidaceae. Although it presents a certain similarity in habit and floral morphology to *Empodium*, *Curculigo* and *Molineria*, none of them has such a distinctive stigma, ovary and seed structure.

**Figure 2:**
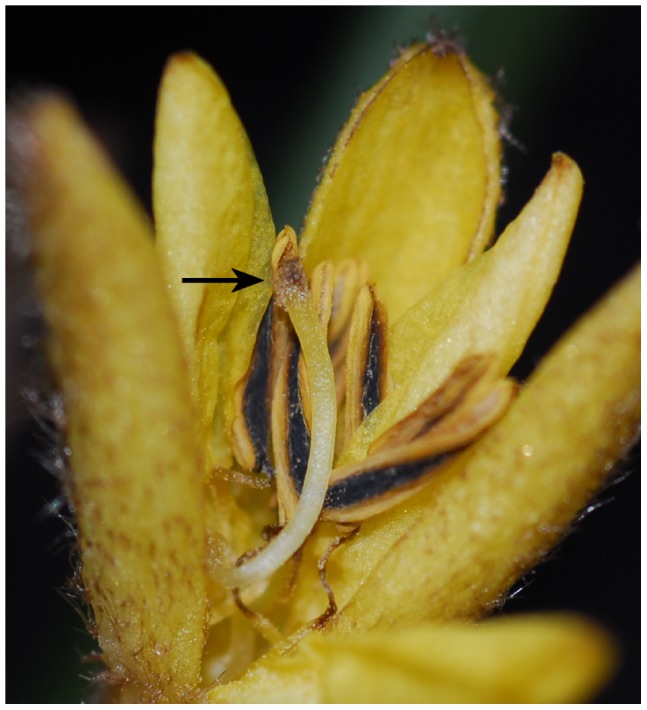
Self-pollination of *Sinocurculigo taishanica.* In the closed flower, the glandular-hairy stigma touches the opened anthers and allows the pollen to adhere, thus facilitating complete pollination (arrow).

### Observation of Pollination Mechanism

The inflorescence of the new plant is characterized by only one flower opening at a time. After the flower is fully open, the perianth segments begin to move back while the style begins to curve at base. In the closed flower, the glandular-hairy stigma touches the opened anthers and allows adherence of the pollen from them in order to complete pollination (Figure 2). The flowers observed have all fruited and borne numerous seeds.

**Table 1:** Statistics from the Family-level analysis datasets.

Information	*rbcL*	*matK*	Combined
No. of taxa	60	58	60
Aligned length	1314	1741	3055
No. variable characters	463	1170	1633
No. informative characters (%)	346 (26.3)	928 (53.3)	1274 (41.7 )
Tree length	1425	5454	5786
Consistency index	0.4442	0.4685	0.4620
Retention index	0.7010	0.7202	0.7149

**Table 2:** Best-fit model and parameter for Family-level analysis datasets.

Region	AIC select model	Base frequencies				substitution model (rate matrix)						I	G
		A	C	G	T	A–C	A–G	A–T	C–G	C–T	G–T		
*rbcL*	GTR+I+G	0.2894	0.1961	0.2245	0.2899	1.2706	3.3681	0.5360	0.9887	4.5414	1.0000	0.4873	0.7629
*matK*	TVM+I+G	0.3302	0.1447	0.1429	0.3821	1.4534	2.3235	0.2661	0.9432	2.3235	1.0000	0.1391	1.9375
Combined	GTR+I+G	0.3173	0.1636	0.1717	0.3474	1.4239	2.4606	0.3375	0.8760	2.7661	1.0000	0.3312	1.4870

### Analyses of Phylogenetic Position

**Figure 3:**
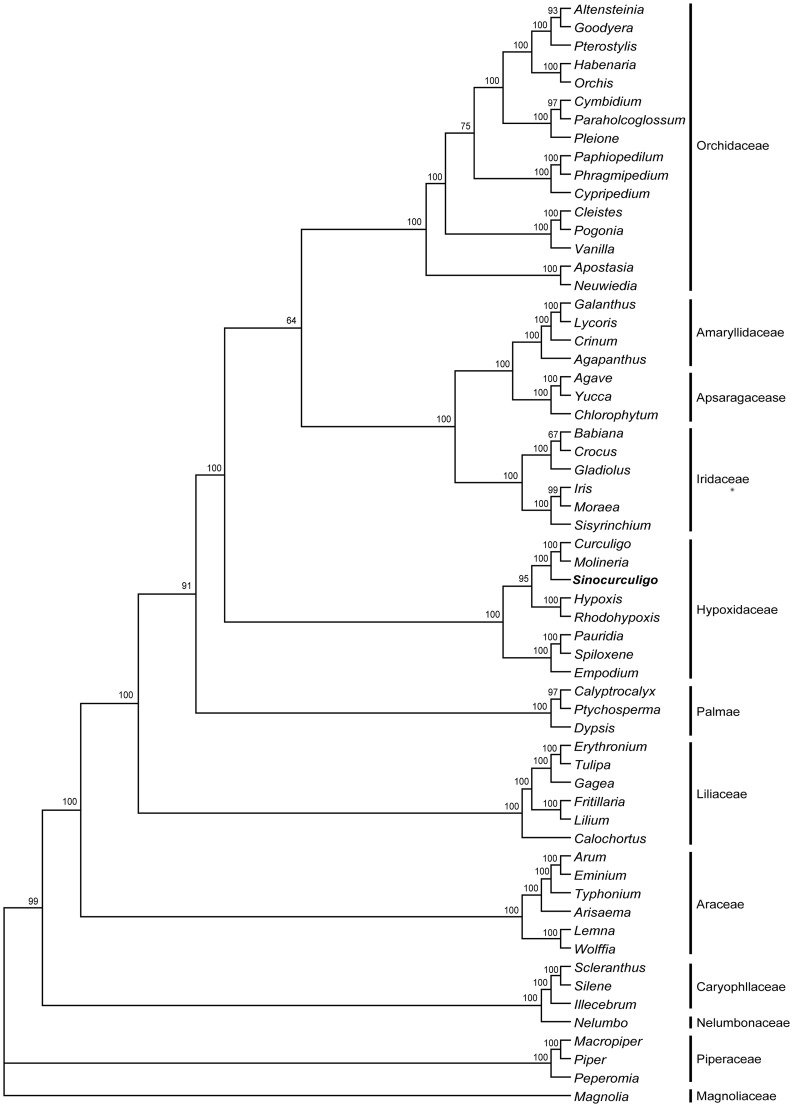
Bayesian tree obtained from analysis of combined dataset of family-level analysis. The Bayesian posterior probability (×100) is indicated above the branches.

A morphological comparison of this hypoxid with the extant members of Hypoxidaceae showed that it is more or less related to *Empodium* and, to a lesser degree, to *Molineria* and *Curculigo* [11], [13]. We integrated a detailed molecular matrix in order to place the plant in an appropriate phylogenetic position. The phylogeny of related families and genera was constructed based on a combined matrix of 3,055 nucleotides sequences of the *rbcL* and *matK* genes of one family of Magnoliopsida, one family of Piperopsida, one family of Caryophyllopsida, 10 families of Liliopsida, and one family of Ranunculopsida, including a total of 60 taxa (Tables S1, S2). The aligned length, the numbers of variable sites and parsimony informative sites, tree statistics for the maximum parsimony (MP) analysis, and the best-fit model selected by Modeltest are presented in Tables 1 and 2. After Bayesian inference of the phylogeny, majority of consensus phylogeny trees demonstrated monophyly of hypoxid plant and the other families with a posterior probability of 64% (Figure 3).

Results indicate that 13 clades were absolutely distinguished, with a posterior probability of 100% (Figure 3 and Figures S2, S3). The 13 clades respectively correspond to 12 families and their evolutionary sequence. Hypoxidaceae is an independent clade (PP100%), in which *Sinocurculigo* is located together with the known genera of this family. Based on the combination of *matK* and *rbcL* gene sequence, the Hypoxidaceae clade is divided into three subclades (PP100%). Individual results of *matK* and *rbcL* are similar to those of their combination (Figures S4, S5, S6, S7, S8, S9).

### Phylogeny of Hypoxidaceae

#### Analysis of single sequence data

Phylogenetic trees based on the analysis of *rbcL* and *trnS*-*G* produced similar topological structures (Figures S10, S11, S12, S13, S14, S15). The trees showed that the 10 genera of this family are grouped into three clades: the first clade (*Pauridia* –*Empodium* clade) includes all the species of *Spiloxene*, *Pauridia*, and *Saniella*, and two species of *Hypoxis* (*Hypoxis glabella and H. occidentalis*); the second clade (*Curculigo* clade) includes *Molineria, Curculigo, Hypoxidia*, and the new genus, *Sinocurculigo*; and the third clade (*Hypoxis* clade) includes most species of *Hypoxis* and *Rhodohyoxis.* The phylogenetic tree of *trnL-F* (Figures S16, S17, S18) showed some differences from those of *rbcL* and *trnS*-*G*. The 10 genera can be grouped into four clades, of which the second and third clades are the same as the above. The first clade can be divided into two distinct clades: the *Pauridia* clade includes *Spiloxene*, *Pauridia*, *Saniella*, and two species of *Hypoxis*; and the genus *Empodium* forms another clade. The three datasets produce rather similar topological structures, especially in the main clades. The aligned length, the numbers of variable sites and parsimony informative sites, tree statistics for the maximum parsimony (MP) analysis, and the best-fit model selected by Modeltest are presented in Tables 3 and 4.

**Table 3:** Statistics from the intra-Hypoxidaceae analysis datasets.

Information	*rbcL*	*trnS*-*G*	*trnL-F*	Combined
No. of taxa	59	56	56	59
Aligned length	1365	1380	1188	3933
No. variable characters	211	536	323	1070
No. informativecharacters (%)	127 (9.3)	294 (21.3)	168 (14.1)	589 (15.0 )
Tree length	330	915	503	1773
Consistency index	0.7273	0.7464	0.7455	0.7321
Retention index	0.8892	0.8179	0.8489	0.8380

**Table 4:** Best-fit model and parameter for intra-Hypoxidaceae analysis datasets.

Region	AIC select model	Base frequencies				substitution model (rate matrix)						I	G
		A	C	G	T	A–C	A–G	A–T	C–G	C–T	G–T		
*rbcL*	TIM+I+G	0.2749	0.2043	0.2412	0.2796	1.0000	1.8162	0.6095	0.6095	3.1561	1.0000	0.6130	0.8764
*trnS*-*G*	K81uf+G	0.3665	0.1341	0.1235	0.3759	1.0000	1.2687	0.4928	0.4928	1.2687	1.0000	0.0000	0.8499
*trnL-F*	TVM+I+G	0.3110	0.1516	0.1520	0.3262	0.9211	1.2446	0.9190	0.2907	1.2446	1.0000	0.3495	0.7340
Combined	TVM+I+G	0.3309	0.1677	0.1699	0.3314	1.0502	1.4812	0.7877	0.4681	1.4812	1.0000	0.4045	0.8171

#### Combined analysis

**Figure 4:**
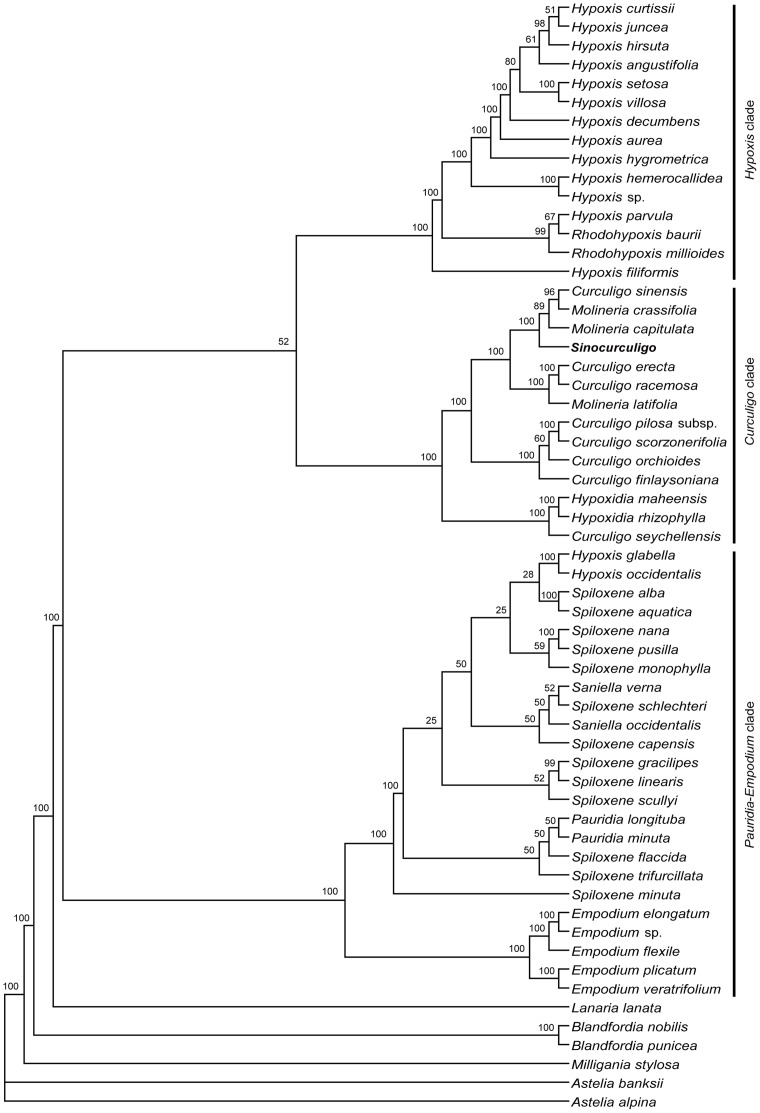
Bayesian tree obtained from the analysis of combined dataset of Hypoxidaceae. The Bayesian posterior probability (×100) is indicated above the branches.

**Figure 5:**
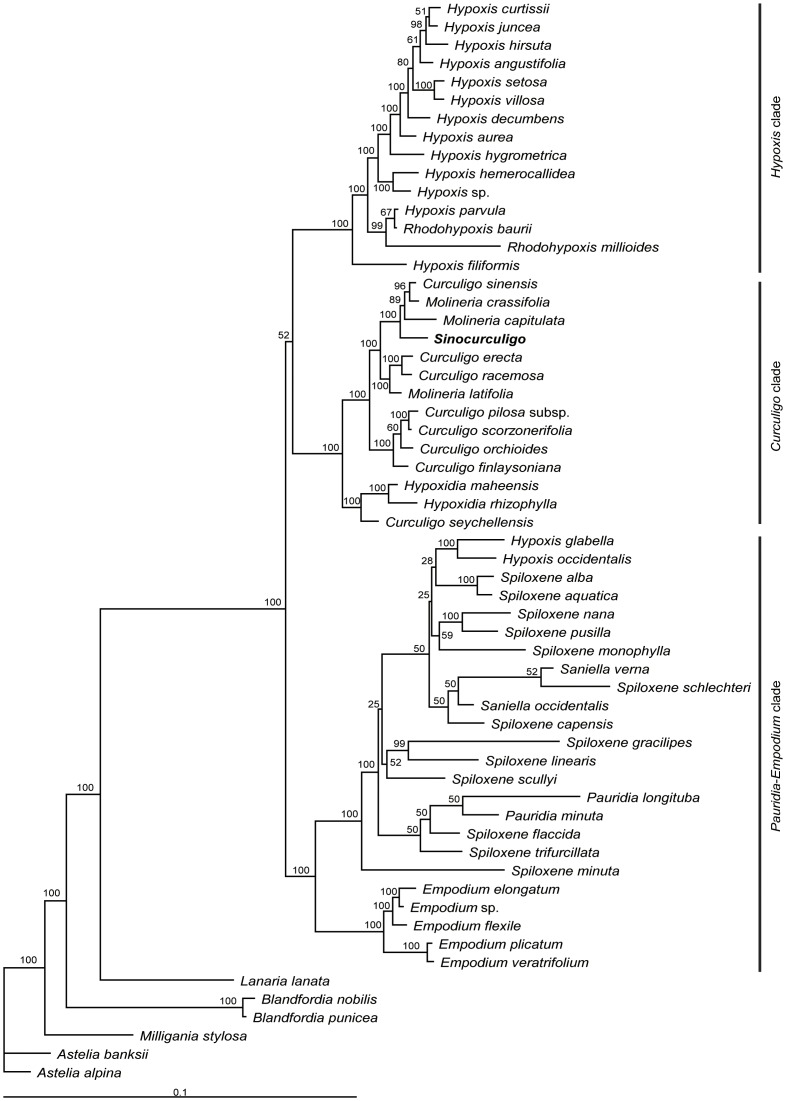
A phylogenetic tree of Bayesian inference consensus trees based on the last 30,001 maximum likelihood trees for combined dataset of Hypoxidaceae. The Bayesian posterior probability (×100) is indicated near the nodes.

In the present study, we combined *rbcL, trnS*-*G*, and *trnL-F* into a single dataset (Figures 4,5 and Figures S19, S20). The strict consensus BI tree (Figures 4, 5) strongly supports the division of Hypoxidaceae into three clades (PP100%). The first clade (southern Africa clade, the *Pauridia-Empodium* clade of Kocyan et al. [7]) consist of *Empodium*, *Spiloxene*, *Pauridia*, *Saniella*, and *Hypoxis* and is strongly supported as a sister to the outgroup clade Lanariaceae (PP100%). The second clade (around Pacific-Indian Ocean clade, the *Curculigo* clade of Kocyan et al. [7]), consisting of *Curculigo, Molineria*, *Hypoxidia*, and *Sinocurculigo*, is weakly supported as a sister to the third clade (PP52%). The third clade (worldwide clade, the *Hypoxis* clade of Kocyan et al. [7]) consists of *Hypoxis* and *Rhodohypoxis*; *Hypoxis* is widespread in Africa, Asia, Australia and Americas, while *Rhodohypoxis* is restricted to southern Africa.

In the around Pacific-Indian Ocean clade, the genus *Sinocurculigo* forms a distinct subclade, being a sister to the subclade containing *Molineria* and *Curculigo*.

## Discussion

To our knowledge, this is the first study to report a member of the family Hypoxidaceae to have heterochlamydeous perianth, elongate filaments arising from style base and adnate to perianth segments with their basal parts, a unique stigma composed of three inverted U-shaped hairy piles, a unilocular ovary with three parietal placentas, and numerous papillate seeds. These features differ sharply from those found in *Empodium*, that is an African genus characterized by having a single ebracteate flower and smooth seeds with a persistent outgrowth on surface [7]; but its allies, *Curculigo* and *Molineria*, have a trilobed stigma, a trilocular ovary, and non-papillate seeds; and in *Hypoxis*, the stigma is usually composed of three lobes or stripes, though in *H. angustifolia* the stigma can differ in shape in different individuals [14]. The glandular-hairy stigma of the Taishan hypoxid is a feature that makes self-fertilization possible, which distinguishes it from all other hypoxids. Findings of this study significantly improve our understanding of the hypoxid evolution in stigma structure and reproductive mechanisms.

Molecular evidence agrees well to the conclusion drawn from morphological features. Analyses of a combined data set with parsimony and Bayesian methods revealed that the Hypoxidaceae is highly monophyletic and that the Taishan hypoxid represents an independent lineage parallel to the genera *Curculigo* and *Molineria* in the family. The Taishan hypoxid is treated as a new genus, *Sinocurculigo*, in the present study.

Our analyses supported the following systematic order of the ten genera: *Curculigo*, *Empodium*, *Hypoxidia*, *Hypoxis*, *Molineria*, *Pauridia*, *Rhodohypoxis*, *Saniella*, *Spiloxene*, and *Sinocurculigo* (Figures 4, 5). These genera, as indicated by Koeyan et al. [7] and Rudall et al. [6], can be grouped into three major clades: (1) around Pacific-Indian Ocean clade, containing *Curculigo*, *Hypoxidia*, *Molineria*, and *Sinocurculigo*; (2) worldwide *Hypoxis* clade, composed of most species *Hypoxis* and *Rhodohypoxis*; and (3) Southern Africa clade, including *Empodium*, a few species of *Hypoxis*, *Pauridia*, *Saniella*, and *Spiloxene*. Among them, *Sinocurculigo* is considered to be a rather advanced genus in Hypoxidaceae.

The flowers of Hypoxidaceae usually use pollen to attract insects to facilitate pollination [7]. Self-pollination has never been found in this family. We have observed honeybees visiting the flowers of *Curculigo sinensis*, *C. orchioides*, and *Molineria capitulata* in Guangdong, but failed to see any insect visiting the flowers of *Sinocurculigo*. *Sinocurculigo* appears to adapt to intra-flower pollination, a derivative mechanism in the family.

Hypoxidaceae has been proposed as a potential sister family to Orchidaceae [15]–[18] based on morphological features. Similar to other members of Hypoxidaceae, *Sinocurculigo* also shows some similarity in leaf and flower characteristics of the two genera of Apostasioideae. Of course, there is a gap between Apostasioideae of Orchidaceae and Hypoxidaceae. The discovery of *Sinocurculigo* at the generic level in the Northern Hemisphere, where *Apostasia* and *Neuwiedia* are abundant, is stirring and very interesting.

### Taxonomic Treatment

#### Sinocurculigo

Z. J. Liu, L. J. Chen et Ke Wei Liu gen. nov. [urn:lsid:ipni.org:names: 77119578–1].

- **Diagnosis.** Genus novum Curculigini et Molineriae simile,a quibus differt stigmate integro glanduloso-hirto, sepalis petalis dissimilibus, filamentis antheris multo longioribus bases styli exorientibus et basibus segmentorum adnatis, ovario uniloculari placentis 3 parietalibus, seminibus dense papillatis.
- The new genus is akin to *Curculigo* and *Molineria*, from which it differs by having an entire glandular-hairy stigma, sepals distinguishable from petals, filaments much longer than anthers and arising from style base and adnate to the base of perianth segments, unilocular ovary with three parietal placentas, and densely papillate seeds.
- **Description.** Rhizomes long. Leaves plicate, basal. Inflorescence erect, short, densely several-flowered; flowers subopposite; pedicel very short; perianth segments 6, in two whorls, free; sepals distinguishable from petals; stamens 6, inserted at base of style; filaments much longer than anthers, adnate to perianth segments with their basal part for 1–2 mm; anthers nearly basifixed; ovary inferior, unilocular with three parietal placentas, without an apical beak; style obscurely 3-ridged, dilated at base, with six rows glandular hairs on its uppermost part and then coming together apically forming an entire, hairy stigma; fruit containing numerous papillate seeds.
- **Type.** *Sinocurculigo taishanica* Z. J. Liu, L. J. Chen et Ke Wei Liu.

#### Sinocurculigo taishanica

**Figure 6:**
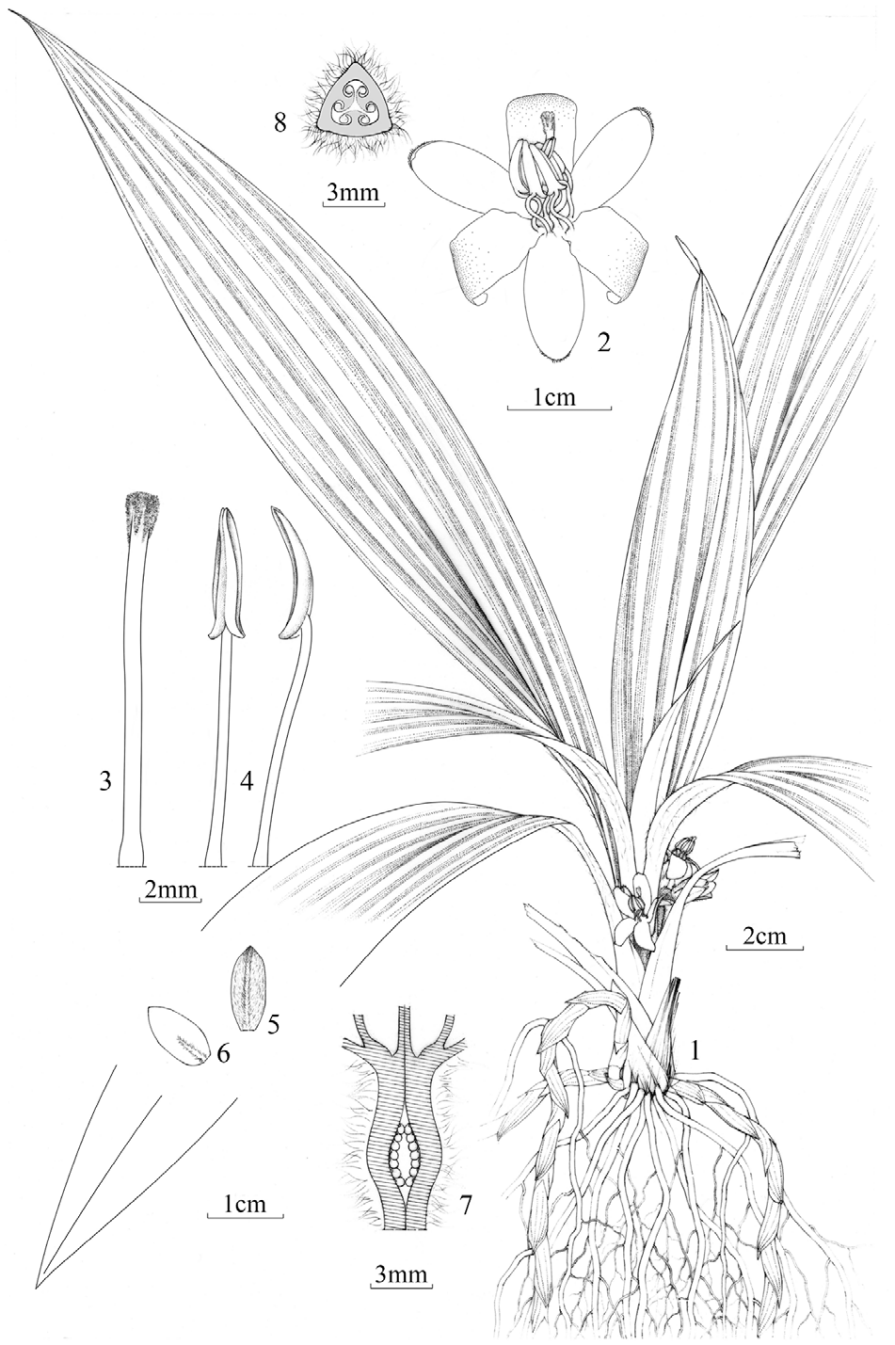
*Sinocurculigo taishanica* Z. J. Liu, L. J. Chen & Ke Wei Liu: **1.** Flowering plant; **2.** Flower, side view; **3.** Stigma, side view; **4.** Anthers, front and side view; **5.** Sepal, back view; **6.** Petal, back view; **7.** Ovary, and base of style, stamens, sepal and petal, longitudinal section; **8.** Ovary, transverse section.

Z. J. Liu, L. J. Chen et Ke Wei Liu sp. nov. [urn:lsid:ipni.org:names: 77119579–1] Figures 1, 6.

- **Type.** Guangdong, Taishan, alt. 450 m. in thicket along a valley, 2011. 11. 28. Z. J. Liu 5829 (NOCC).
- **Etymology.** The generic name *Sinocurculigo* derives from China (Sino) and *Curculigo* (a genus of Hypoxidaceae), referring to Chinese *Curculigo*; *taishanica* is named after the locality (Taishan city).
- **Diagnosis.** same as the genus.
- [LOOSEST]**Description.** Terrestrial herbs. Leaves basal, 6–7, narrowly elliptic-lanceolate, chartaceous, 22–29 cm long, 3.3–4.5 cm wide, apex long-acuminate, subsessile or shortly petioled, tomentose-villose basally. Scape axillary, 3.5–4.5 cm long; peduncle and rachis brown-tomentose; inflorescence erect, racemose, with 4–6 flowers; flowers subopposite, yellow, opening successively; bracts oblong-lanceolate, 1.2–1.9 cm long, acuminate at apex, tomentose-villose abaxially, ciliate; pedicel very short; ovary 0.7–0.9 cm long, densely brown-tomentose, unilocular; sepals oblong, 1.1–1.3 cm long, 5–5.5 mm wide, subobtuse at apex, villose abaxially; petals elliptic-oblong, 1.1–1.2 cm long, 5.5–6 mm wide, slightly crisped-margined, obtuse at apex, villose along abaxial midvein; filaments 8–10 mm long, adnate to the perianth segments at base for 1–2 mm; anthers narrowly lanceolate, 4.3–4.6 mm long, base bilobed; style terete, obscurely 3-ridged, 1.1–1.2 cm long, base dilated, terminating in an entire stigma, represented by a hairy apex and six rows of hairs below the apex; seeds subglobose, papillate.
- **Flowering period.** November to December.
- **Distribution.** China, Guangdong, Taishan city.
- **Habitat.** In thickets along a valley at an elevation between 400 and 500 m and in the forest, accompanying plants consisting of *Gordonia axillaris* (Roxb.) Dietr. (Theaceae), *Enkianthus quinqueflorus* Lour. (Ericaceae), and *Indocalamus longiauritus* Hand.-Mazz. (Bambusaceae); associated with orchids *Apostasia shenzhenian* Z. J. Liu et L. J. Chen [19], *Cymbidium sinense* (Jackson ex Andr.) Willd., and *C. ensifolium* (L.) Sw. [20] (Figure S1).

## Materials and Methods

All necessary permits for our field studies were obtained. The locations for our field studies are not private lands but protected areas controlled by the Forestry Bureau of Guangdong Province, China. We have obtained a valid permit from this authoritative organization. Field observations did not damage any plant, animal, or insect. Although the species of Hypoxidaceae are not endangered plants, *Sinocurculigo taishanica* is a rare plant facing threat and needing protection.

### Materials

A total 53 species in ten genera of Hypoxidaceae were analyzed and six representatives of Asteliaceae, Blandfordiaceae, and Lanariaceae were chosen as outgroup. The new hypoxid was sampled in this study and others were accessed from GenBank. In addition, 60 genera representing 12 closely related families (e.g. Orchidaceae, Palmas, Nelumbonaceae, Amaryllidaceae, Asparagaceae, Liliaceae, Caryophyllaceae, Piperaceae, Araceae, and Iridaceae) were accessed from GenBank and were treated as an ingroup in order to test the monophyly of Hypoxidaceae and to interpret its genetic relationships. One species of Magnoliaceae was chosen as outgroup [21], [22]. For detailed information regarding the assessment, see Supplementary Tables S1 and S2.

### Amplification and Sequencing

Total DNA was extracted from fresh material or silica-gel-dried plant tissue with a Multisource Genomic DNA Miniprep Kit (Axygen Biosciences) following the manufacturer’s instructions. The amplification reaction included total DNA, primers, Ex-Taq buffer, and Ex-Taq DNA polymerase (Takara Bio). The polymerase chain reaction (PCR) profile consisted of an initial 5 min pre-melt stage at 95°C, then 30 cycles of 30 s at 95°C (denaturation), 30 s at 46–52°C (annealing temperature was determined by primer), and 1–3 min at 72°C (extension time was determined by length of the target DNA region), followed by a final 10-min extension at 72°C.

Amplification of the *rbcL* region was performed using the primer pairs *rbcL-1F* and *rbcL*-1352R [23]. The *trnL-F* region was amplified with primers c and f [24]. For *matK* sequences, amplification was performed using the primer pair *matK*-*19F* and *trnK*-*2R* [25]. The spacer region between *trnS*-*trnG* was amplified using the S and G primers [26]. To check the quality of the amplified DNA, PCR products were run on 1.5% agarose gels. Gels with target products were excised, purified using DNA Gel Extraction Kits (Axygen Biosciences), and sequenced by BGI Americas Corporation.

#### Sequence editing and assembly

Both forward and reverse sequences and electropherograms were edited and assembled with DNASTAR (http://www.dnastar.com/). DNA sequences were aligned with MEGA5.05 [27] under the Muscle model and then adjusted manually with MEGA5.05 [27]. Aligned sequences are available from the corresponding author upon request.

#### Data analyses

For the family-level analysis, the datasets included *rbcL, matK*, and their combination. Under the family analysis, the datasets included *rbcL*, *trnS-G*, *trnL-F*, and their combination. Insertions, deletions, and some unavailable sequences were treated as missing. Phylogenetic analyses were performed under maximum likelihood (ML), maximum parsimony (MP), and Bayesian inference (BI). The best fit model for each dataset was selected by Modeltest 3.7 [28] under the Akaike Information Criterion (AIC) (Tables S1, S2).

MP analyses were performed using PAUP* version 4.0b10 [29]. All characters were equally weighed and unordered. Test settings included 1,000 replications of random addition sequence and heuristic search with tree bisection-reconnection (TBR) branch swapping. Tree length, consistency indices (CI), and retention indices (RI) are given in Table 1. The ML analysis was computed by RAxML version 7.2.8 with 100 bootstrap replicates using settings described by Stamatakis et al. [30]. BI analysis was performed using MrBayes 3.1.2 [31]. The best-fit model for each dataset was selected by Modeltest 3.7. In the combined datasets, the models were based on the best fit model for each individual dataset. The following settings were applied: sampling frequency  = 100; temp  = 0.1; burn-in  = 10,000; and the number of Markov chain Monte Carlo (MCMC) generations  = 4,000,000. The first 10,000 trees were discarded as burn-in to ensure that the chains are stationary. Majority-rule consensus tree was constructed on those trees sampled after generation 1,000,000.

### Nomenclature

The electronic version of this article in Portable Document Format (PDF) in a work with an ISSN or ISBN will represent a published work according to the International Code of Nomenclature for algae, fungi, and plants, and hence the new names contained in the electronic publication of a *PLoS ONE* article are effectively published under that Code from the electronic edition alone, so there is no longer any need to provide printed copies.

In addition, new names contained in this work have been submitted to IPNI, from where they will be made available to the Global Names Index. The IPNI LSIDs can be resolved and the associated information viewed through any standard web browser by appending the LSID contained in this publication to the prefix http://ipni.org/. The online version of this work is archived and available from the following digital repositories: PubMed Central, LOCKSS.

## Supporting Information

Figure S1 ***Sinocurculigo taishanica*** **.** A. Natural habitat in type locality; B. Growth in thickets.
(TIF)

Figure S2 **Maximum likelihood (ML) trees of combined dataset of family-level analysis computed by RAxML with 100 bootstrap replicates.** Bootstrap values are indicated above the branches.
(TIF)

Figure S3 **Strict consensus tree of the most parsimonious trees based on combined dataset of family-level analysis.** Bootstrap values of the maximum parsimony analysis are indicated above the branches.
(TIF)

Figure S4 **Bayesian tree obtained from the analysis of** ***matK*** **dataset of family-level analysis.** The Bayesian posterior probability (×100) is specified above the branches.
(TIF)

Figure S5 **Maximum likelihood (ML) trees of** ***matK*** **dataset of family-level analysis computed by RAxML with 100 bootstrap replicates.** Bootstrap values are indicated above the branches.
(TIF)

Figure S6 **Strict consensus tree of the most parsimonious trees based on** ***matK*** **dataset of family-level analysis.** Bootstrap values of the maximum parsimony analysis are indicated above the branches.
(TIF)

Figure S7 **Bayesian tree obtained from the analysis of** ***rbcL*** **dataset of family-level analysis.** The Bayesian posterior probability (×100) is indicated above the branches.
(TIF)

Figure S8 **Maximum likelihood (ML) trees of** ***rbcL*** **dataset of family-level analysis computed by RAxML with 100 bootstrap replicates.** Bootstrap values are indicated above the branches.
(TIF)

Figure S9 **Strict consensus tree of the most parsimonious trees based on** ***rbcL*** **dataset of family-level analysis.** Bootstrap values of the maximum parsimony analysis are indicated above the branches.
(TIF)

Figure S10 **Bayesian tree obtained from the analysis of** ***rbcL*** **dataset of Hypoxidaceae.** The Bayesian posterior probability (×100) is indicated above the branches.
(TIF)

Figure S11 **Maximum likelihood (ML) trees of** ***rbcL*** **dataset of Hypoxidaceae computed by RAxML with 100 bootstrap replicates.** Bootstrap values are indicated above the branches.
(TIF)

Figure S12 **Strict consensus tree of the most parsimonious trees based on** ***rbcL*** **dataset of Hypoxidaceae.** Bootstrap values of the maximum parsimony analysis are indicated above the branches.
(TIF)

Figure S13 **Bayesian tree obtained from the analysis of** ***trnS*** **-** ***G*** **dataset of Hypoxidaceae.** The Bayesian posterior probability (×100) is indicated above the branches.
(TIF)

Figure S14 **Maximum likelihood (ML) trees of** ***trnS*** **-** ***G*** **dataset of Hypoxidaceae, computed by RAxML with 100 bootstrap replicates.** Bootstrap values are indicated above the branches.
(TIF)

Figure S15 **Strict consensus tree of the most parsimonious trees based on** ***trnS*** **-** ***G*** **dataset of Hypoxidaceae.** Bootstrap values of the maximum parsimony analysis are indicated above the branches.
(TIF)

Figure S16 **Bayesian tree obtained from the analysis of** ***trnL*** **-** ***F*** **dataset of Hypoxidaceae.** The Bayesian posterior probability (×100) is indicated above the branches.
(TIF)

Figure S17 **Maximum likelihood (ML) trees of** ***trnL*** **-** ***F*** **dataset of Hypoxidaceae, computed by RAxML with 100 bootstrap replicates.** The bootstrap values are indicated above the branches.
(TIF)

Figure S18 **Strict consensus tree of the most parsimonious trees based on** ***trnL*** **-** ***F*** **dataset of Hypoxidaceae.** The bootstrap values of the maximum parsimony analysis are indicated above the branches.
(TIF)

Figure S19 **Maximum likelihood (ML) trees of combined dataset of Hypoxidaceae, computed by RAxML with 100 bootstrap replicates.** Bootstrap values are indicated above the branches.
(TIF)

Figure S20 **Strict consensus tree of the most parsimonious trees based on combined dataset of Hypoxidaceae.** The bootstrap values of the maximum parsimony analysis are indicated above the branches.
(TIF)

Table S1 Species and gene regions sequenced for family-level analysis in this study.
(DOC)

Table S2 Species and gene regions sequenced for intra-Hypoxidaceae analysis and GenBank accession numbers.
(DOC)
