# Supplementary figures and images for: Sinocurculigo, a New Genus of Hypoxidaceae from China Based on Molecular and Morphological Evidence
Source: PLoS One. 2012 Jun 27;7(6):e38880. doi: 10.1371/journal.pone.0038880 (PMC3384634; doi:10.1371/journal.pone.0038880)

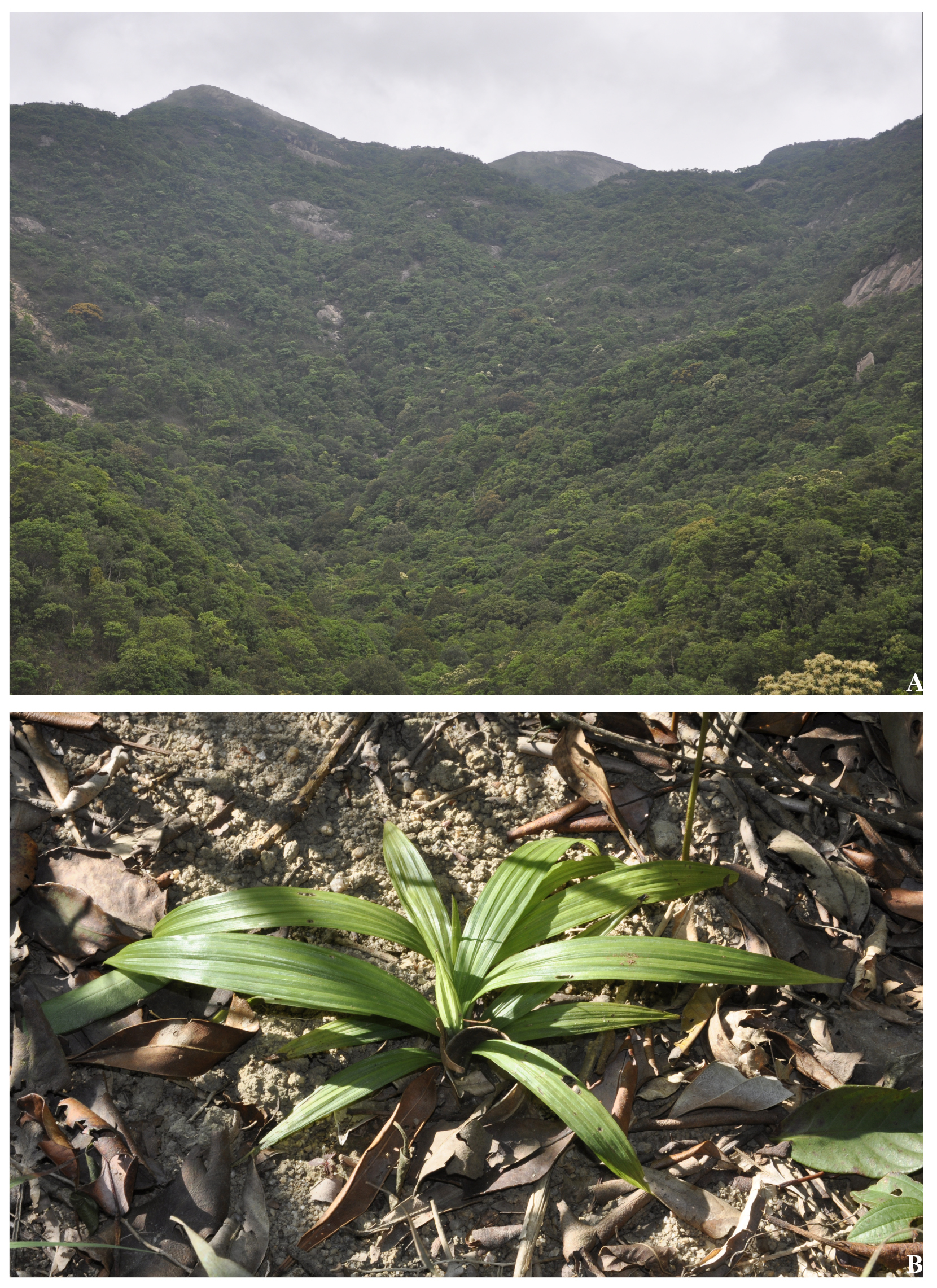

Supplement: Figure S1 — Sinocurculigo taishanica . A. Natural habitat in type locality; B. Growth in thickets. (TIF) [file pone.0038880.s001.tif]

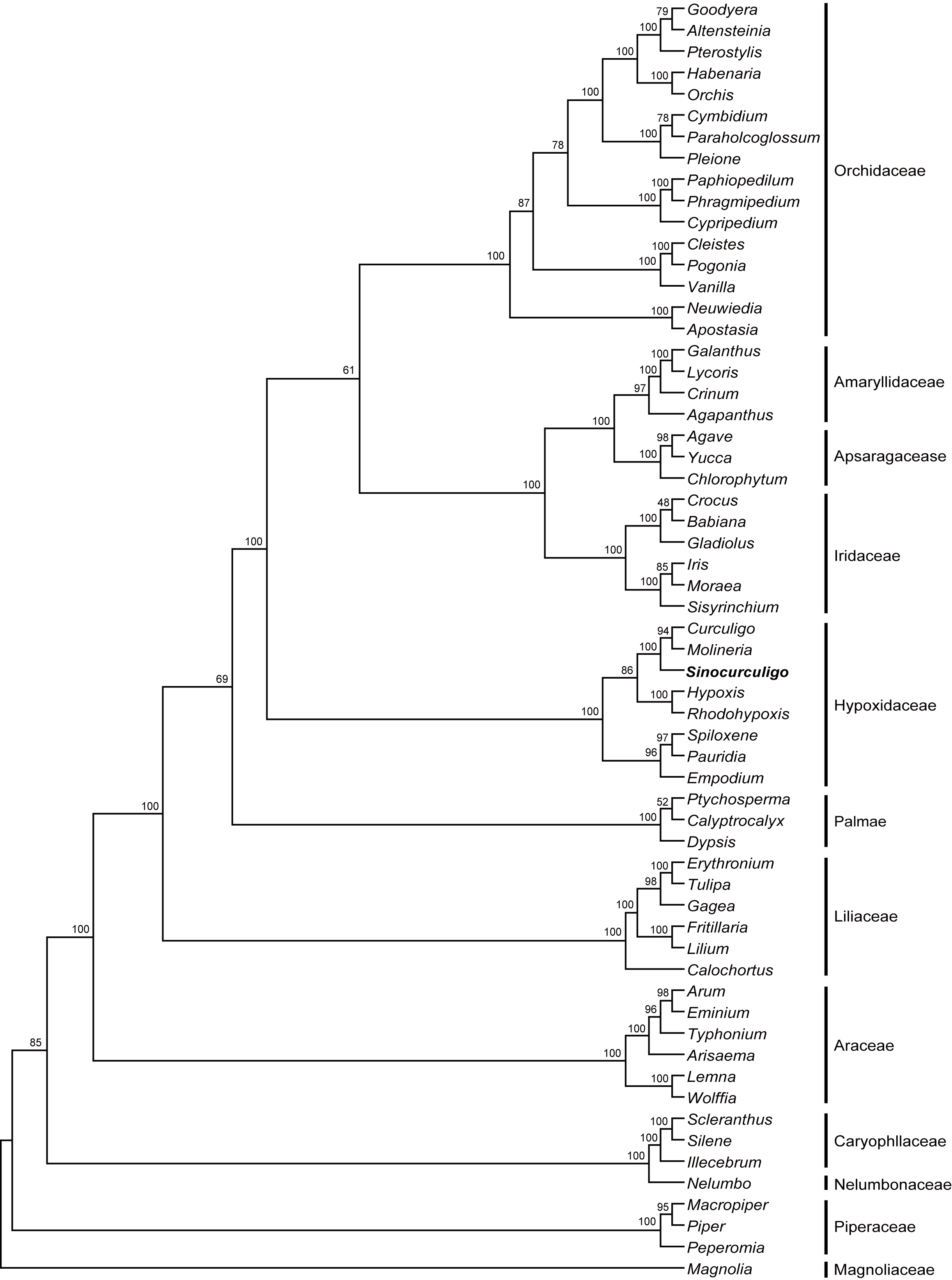

Supplement: Figure S2 — Maximum likelihood (ML) trees of combined dataset of family-level analysis computed by RAxML with 100 bootstrap replicates. Bootstrap values are indicated above the branches. (TIF) [file pone.0038880.s002.tif]

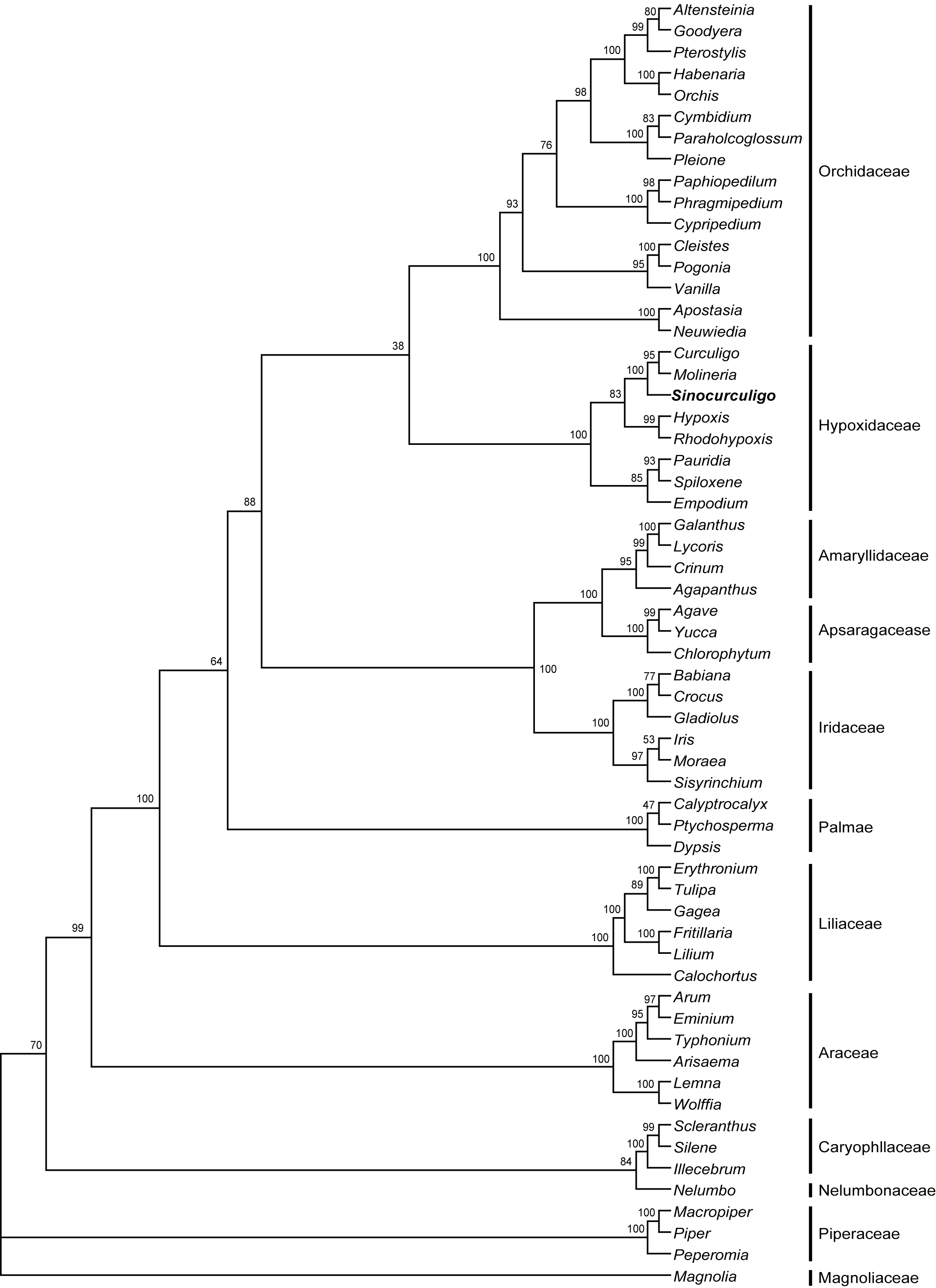

Supplement: Figure S3 — Strict consensus tree of the most parsimonious trees based on combined dataset of family-level analysis. Bootstrap values of the maximum parsimony analysis are indicated above the branches. (TIF) [file pone.0038880.s003.tif]

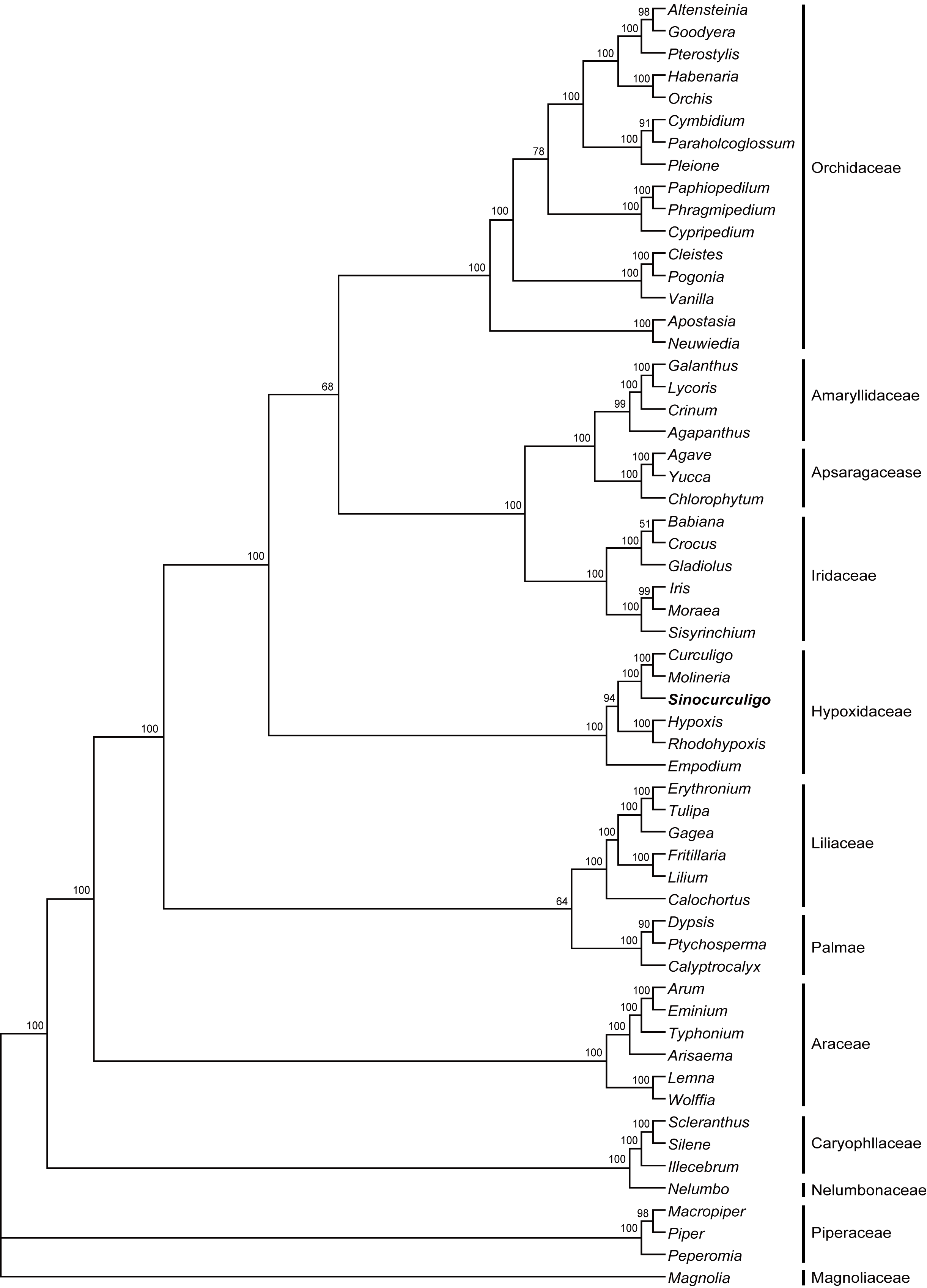

Supplement: Figure S4 — Bayesian tree obtained from the analysis of matK dataset of family-level analysis. The Bayesian posterior probability (×100) is specified above the branches. (TIF) [file pone.0038880.s004.tif]

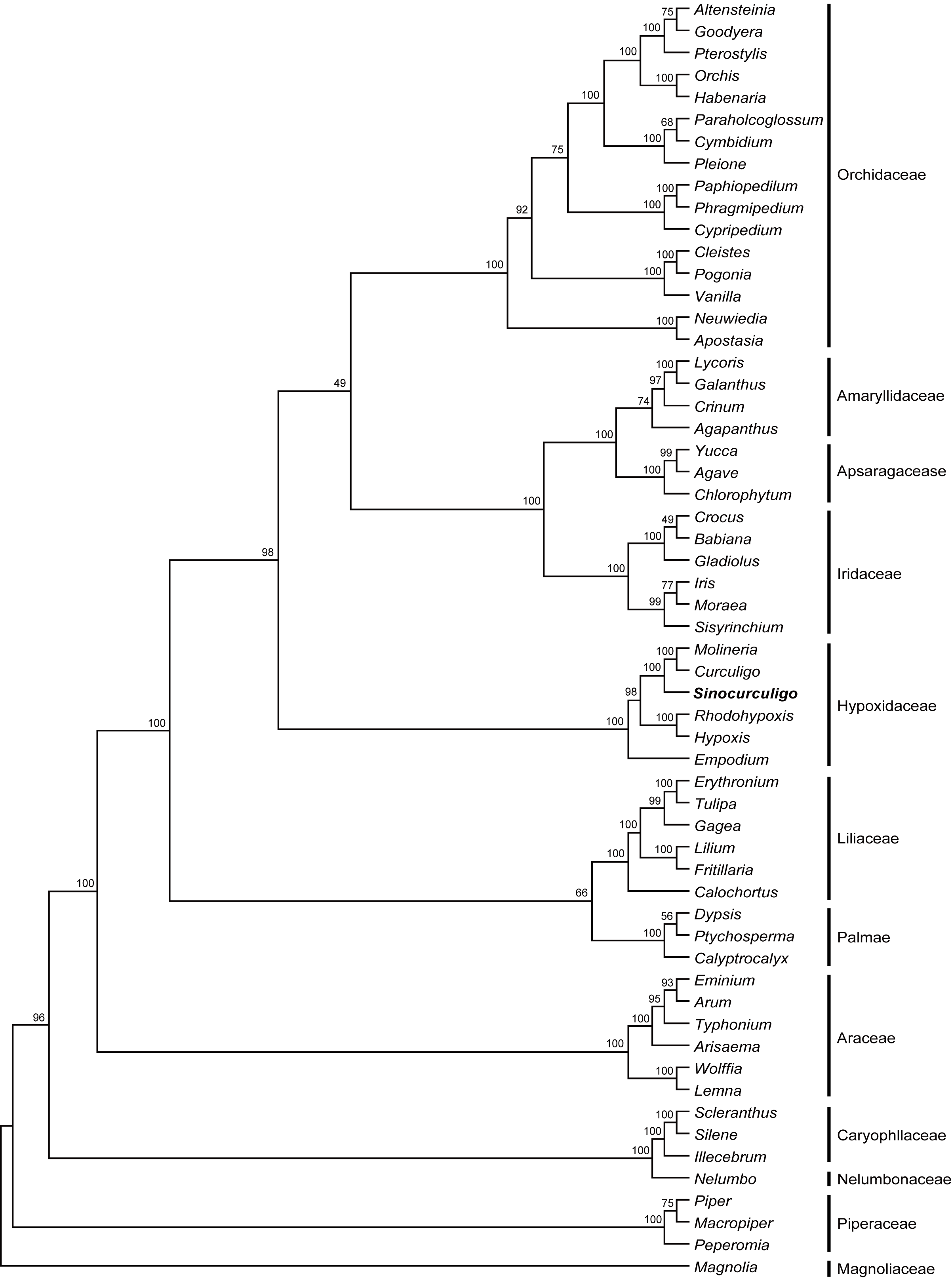

Supplement: Figure S5 — Maximum likelihood (ML) trees of matK dataset of family-level analysis computed by RAxML with 100 bootstrap replicates. Bootstrap values are indicated above the branches. (TIF) [file pone.0038880.s005.tif]

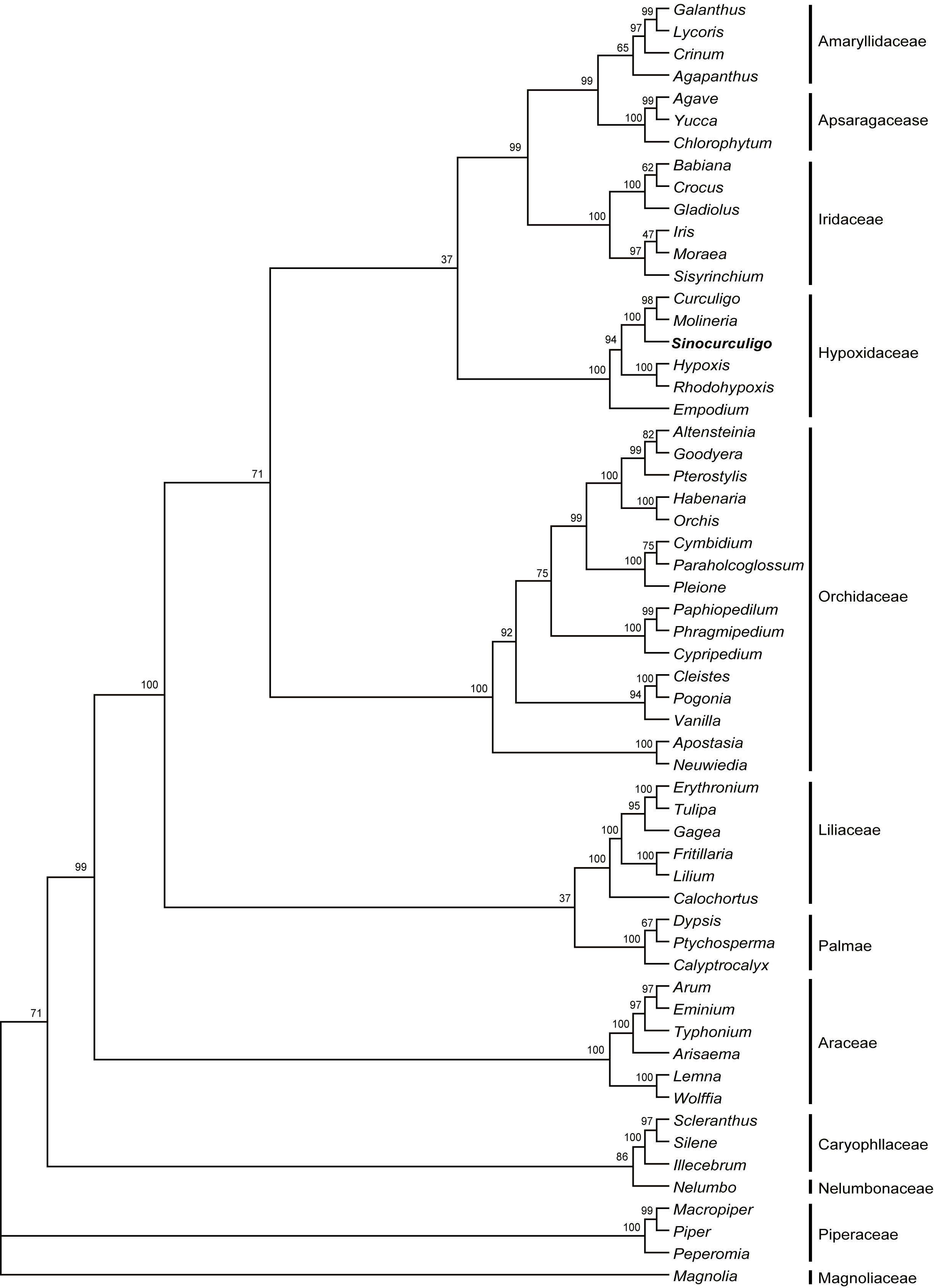

Supplement: Figure S6 — Strict consensus tree of the most parsimonious trees based on matK dataset of family-level analysis. Bootstrap values of the maximum parsimony analysis are indicated above the branches. (TIF) [file pone.0038880.s006.tif]

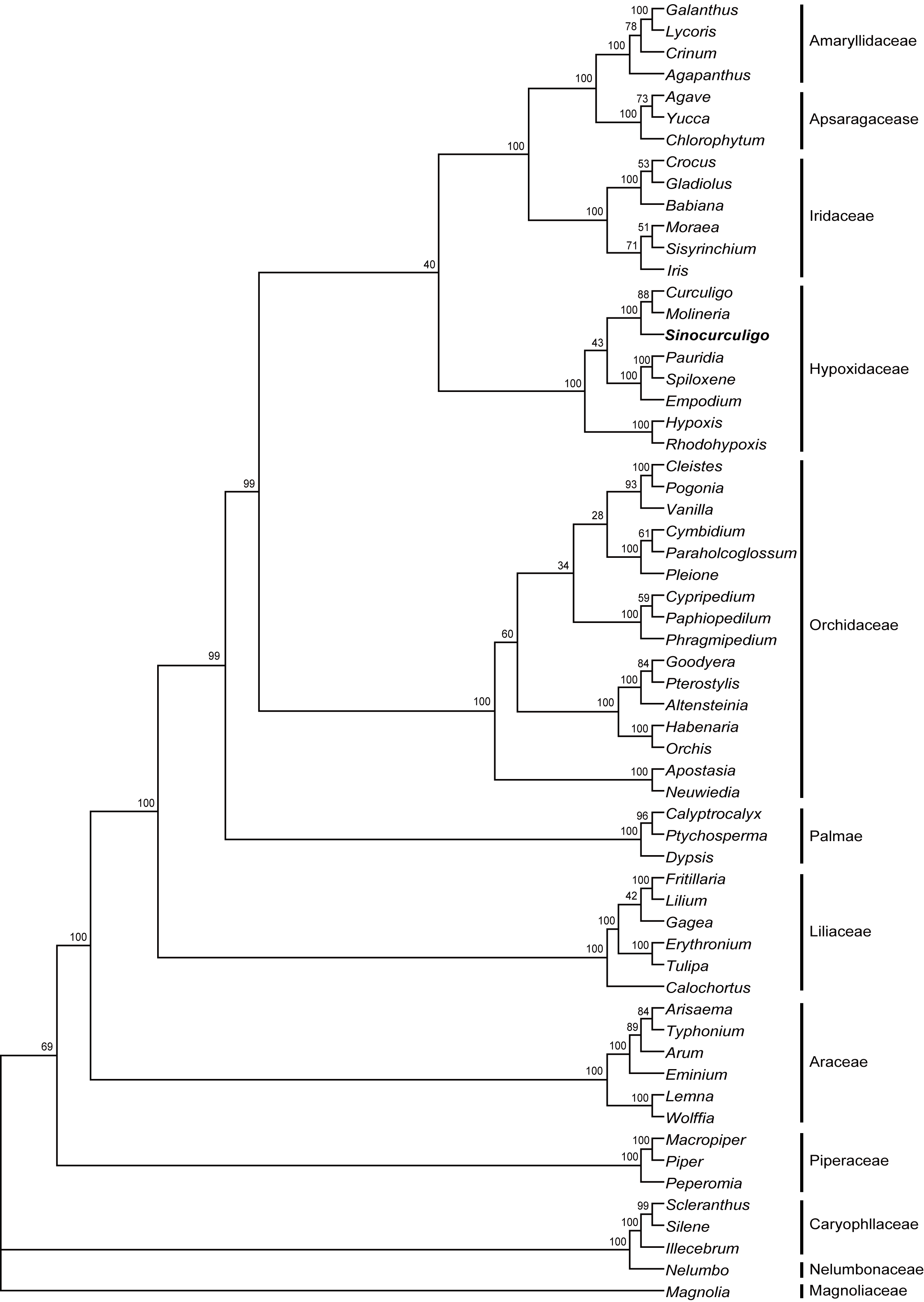

Supplement: Figure S7 — Bayesian tree obtained from the analysis of rbcL dataset of family-level analysis. The Bayesian posterior probability (×100) is indicated above the branches. (TIF) [file pone.0038880.s007.tif]

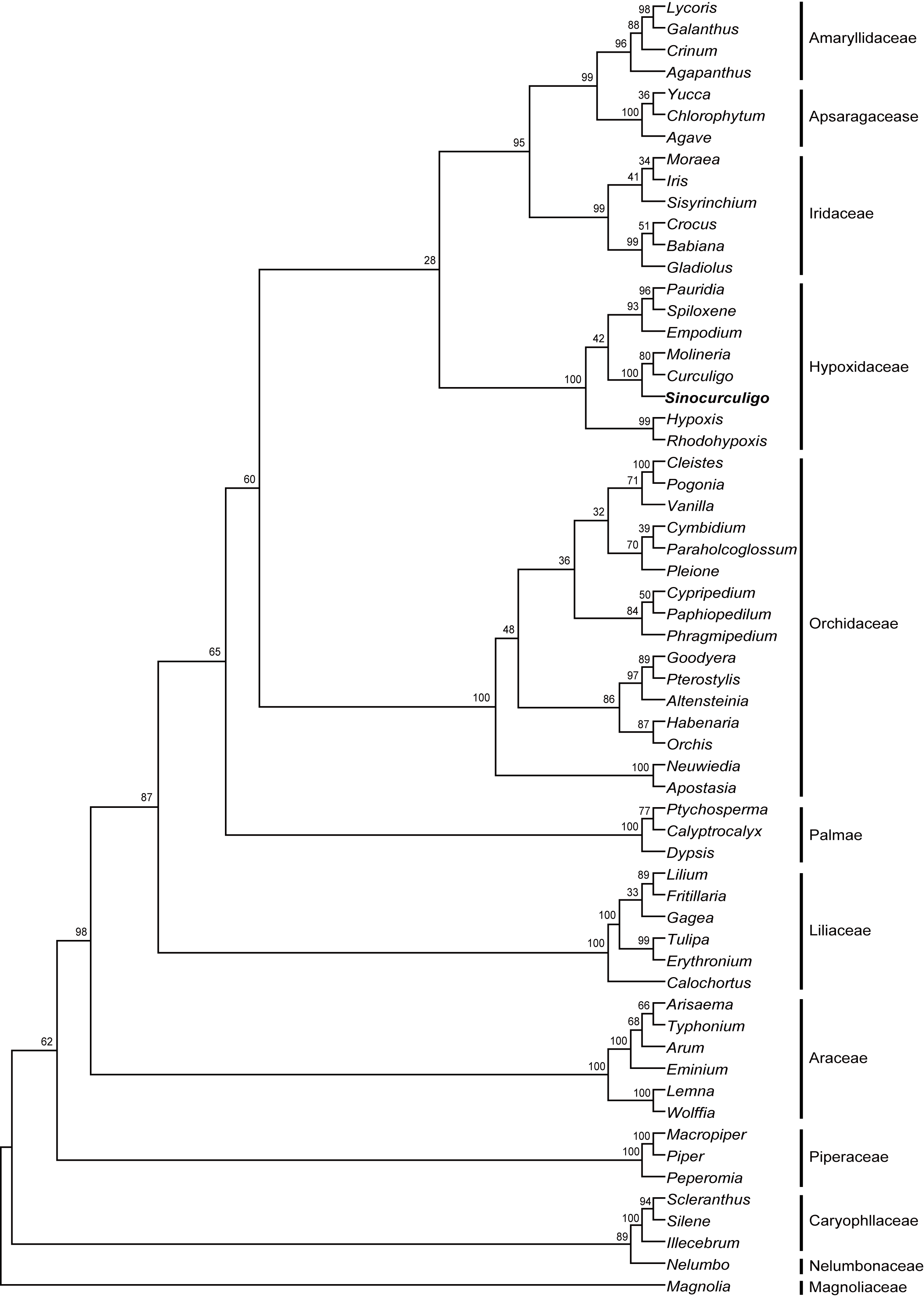

Supplement: Figure S8 — Maximum likelihood (ML) trees of rbcL dataset of family-level analysis computed by RAxML with 100 bootstrap replicates. Bootstrap values are indicated above the branches. (TIF) [file pone.0038880.s008.tif]

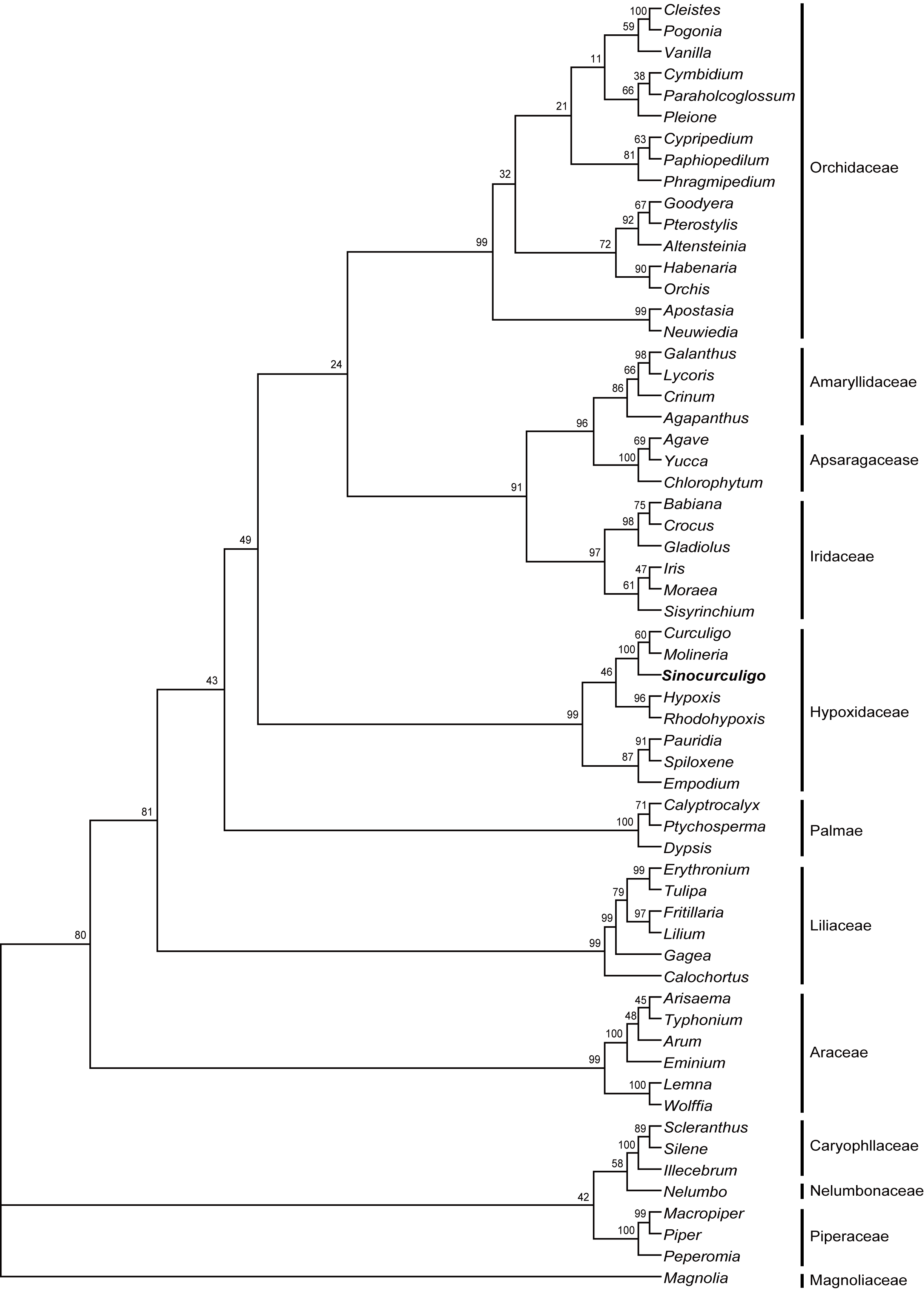

Supplement: Figure S9 — Strict consensus tree of the most parsimonious trees based on rbcL dataset of family-level analysis. Bootstrap values of the maximum parsimony analysis are indicated above the branches. (TIF) [file pone.0038880.s009.tif]

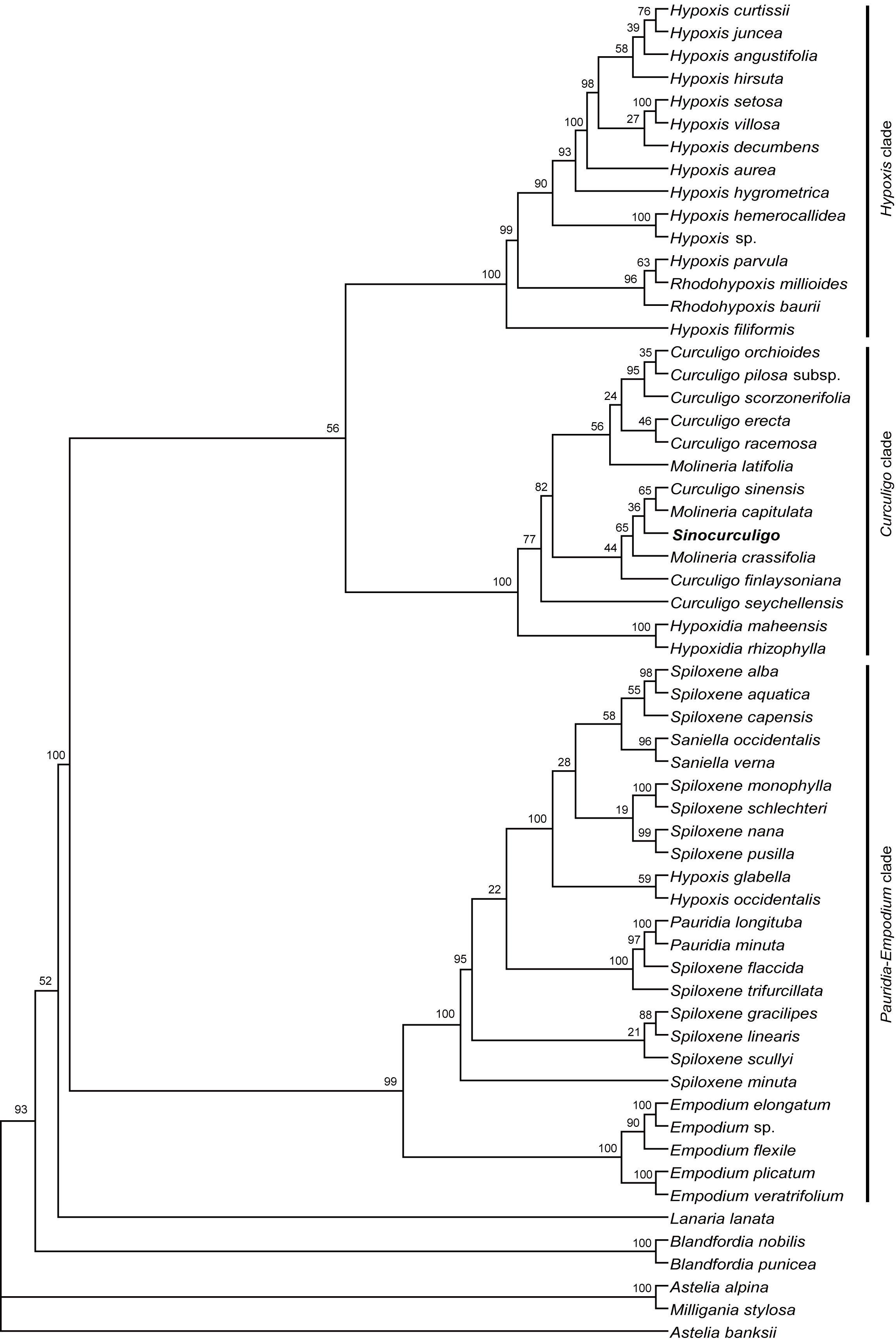

Supplement: Figure S10 — Bayesian tree obtained from the analysis of rbcL dataset of Hypoxidaceae. The Bayesian posterior probability (×100) is indicated above the branches. (TIF) [file pone.0038880.s010.tif]

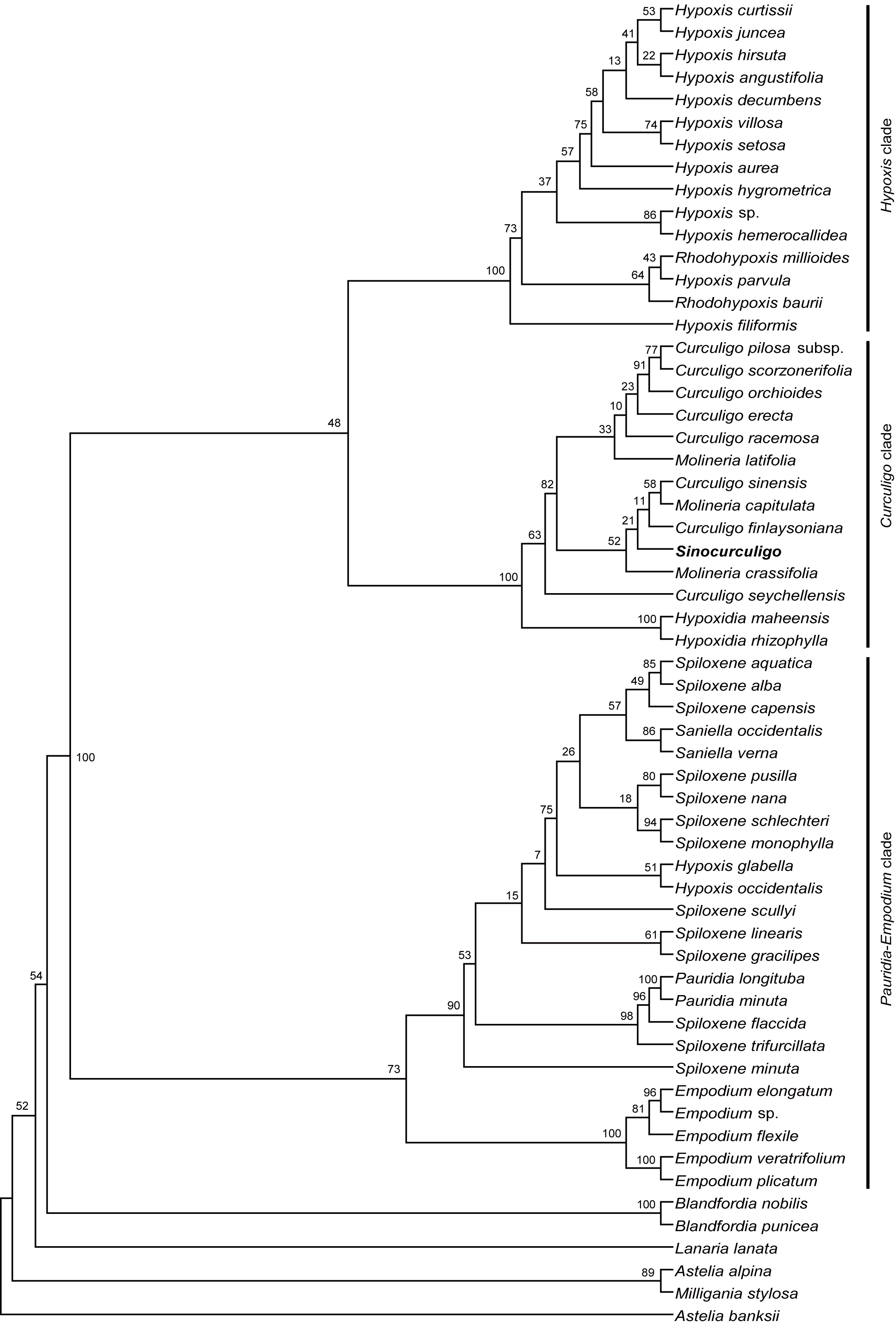

Supplement: Figure S11 — Maximum likelihood (ML) trees of rbcL dataset of Hypoxidaceae computed by RAxML with 100 bootstrap replicates. Bootstrap values are indicated above the branches. (TIF) [file pone.0038880.s011.tif]

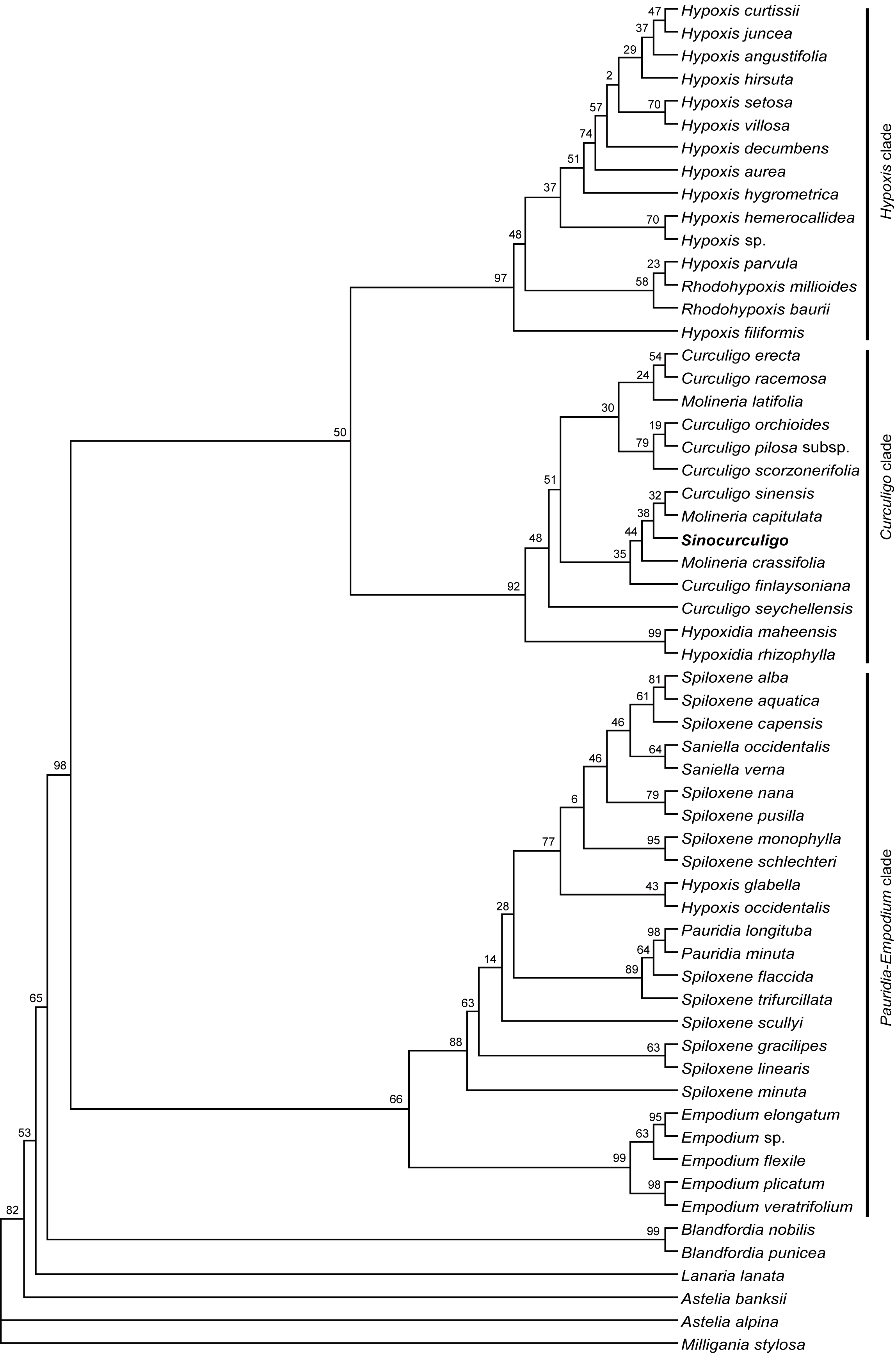

Supplement: Figure S12 — Strict consensus tree of the most parsimonious trees based on rbcL dataset of Hypoxidaceae. Bootstrap values of the maximum parsimony analysis are indicated above the branches. (TIF) [file pone.0038880.s012.tif]

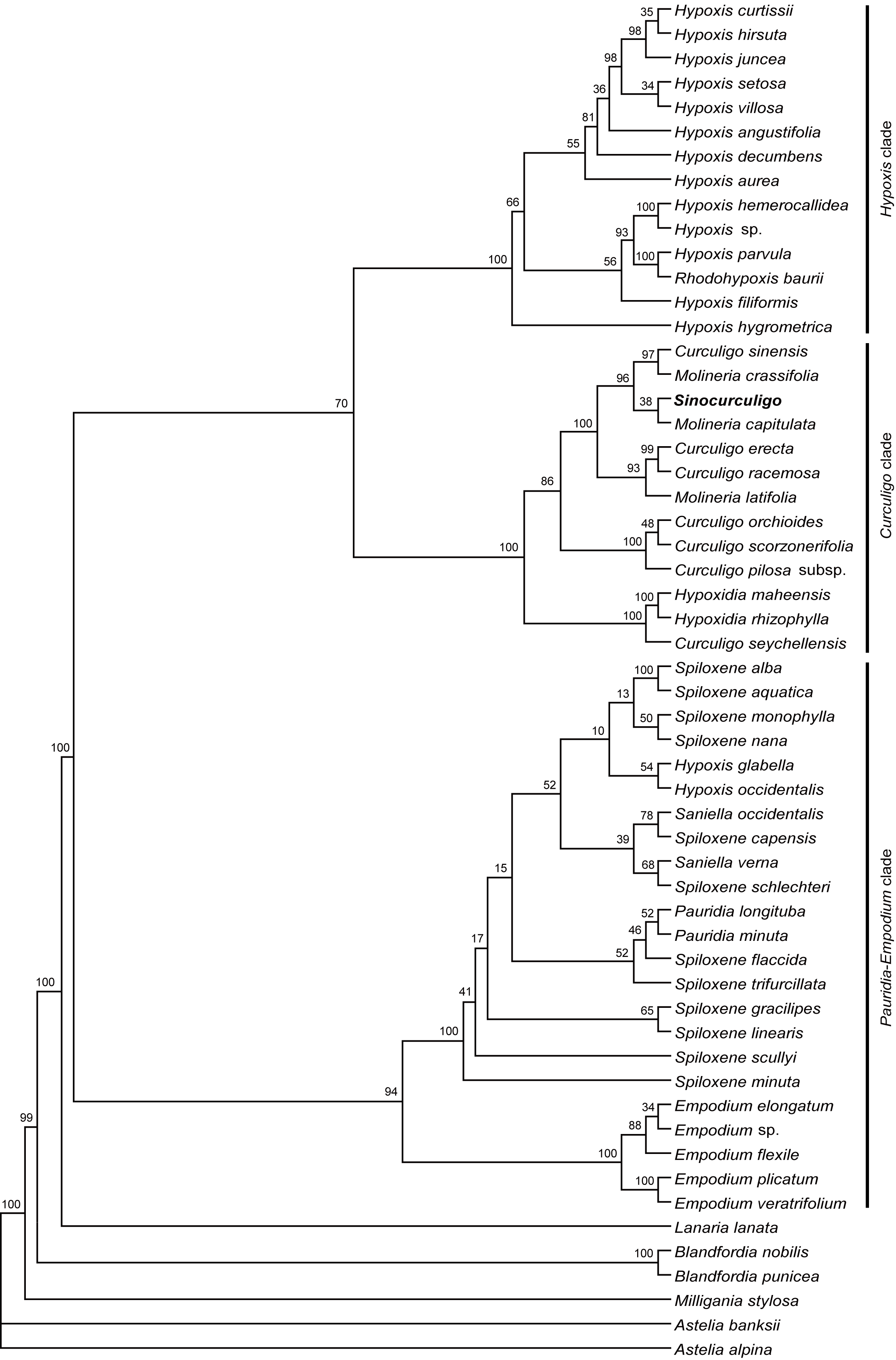

Supplement: Figure S13 — Bayesian tree obtained from the analysis of trnS - G dataset of Hypoxidaceae. The Bayesian posterior probability (×100) is indicated above the branches. (TIF) [file pone.0038880.s013.tif]

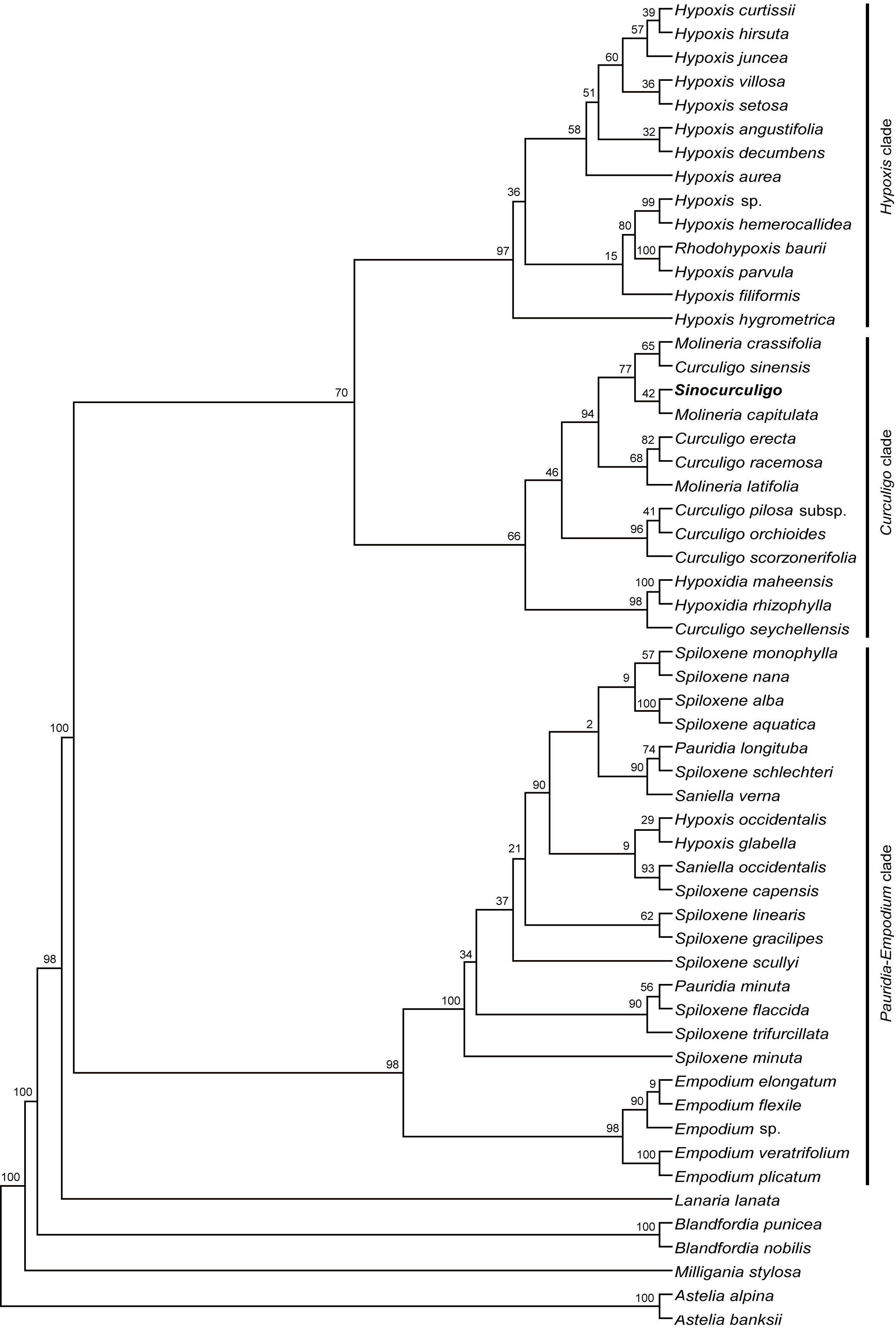

Supplement: Figure S14 — Maximum likelihood (ML) trees of trnS - G dataset of Hypoxidaceae, computed by RAxML with 100 bootstrap replicates. Bootstrap values are indicated above the branches. (TIF) [file pone.0038880.s014.tif]

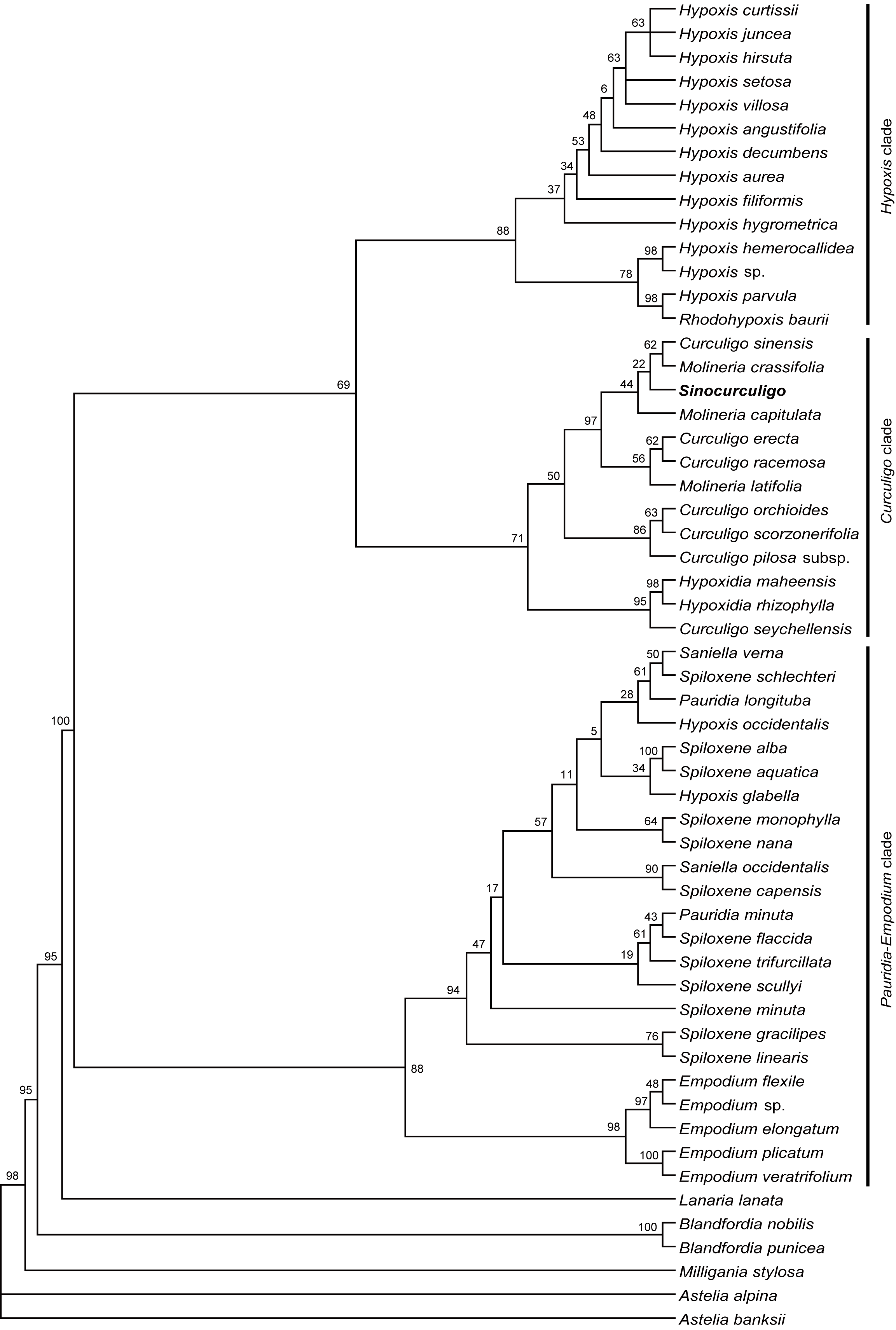

Supplement: Figure S15 — Strict consensus tree of the most parsimonious trees based on trnS - G dataset of Hypoxidaceae. Bootstrap values of the maximum parsimony analysis are indicated above the branches. (TIF) [file pone.0038880.s015.tif]

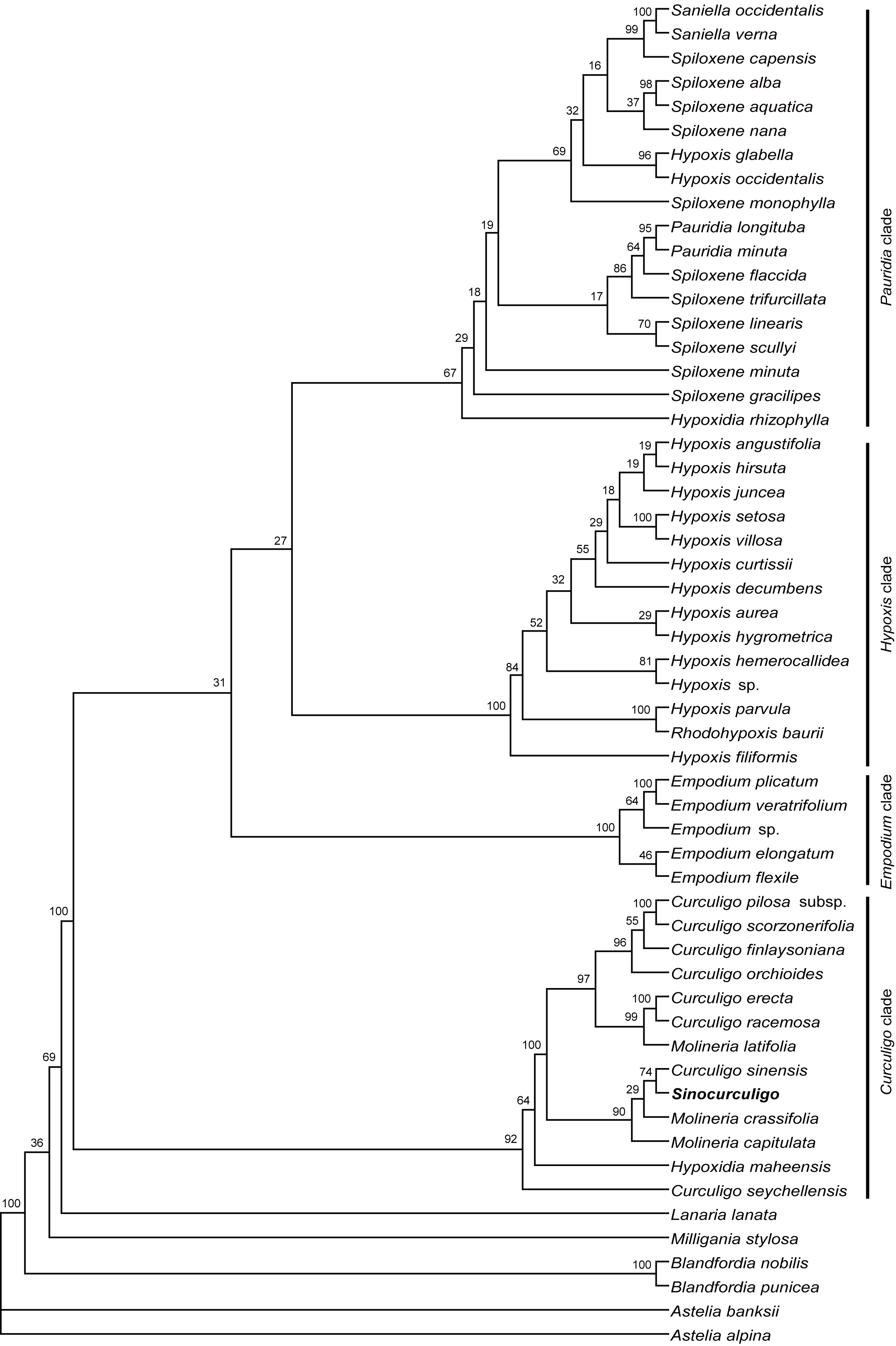

Supplement: Figure S16 — Bayesian tree obtained from the analysis of trnL - F dataset of Hypoxidaceae. The Bayesian posterior probability (×100) is indicated above the branches. (TIF) [file pone.0038880.s016.tif]

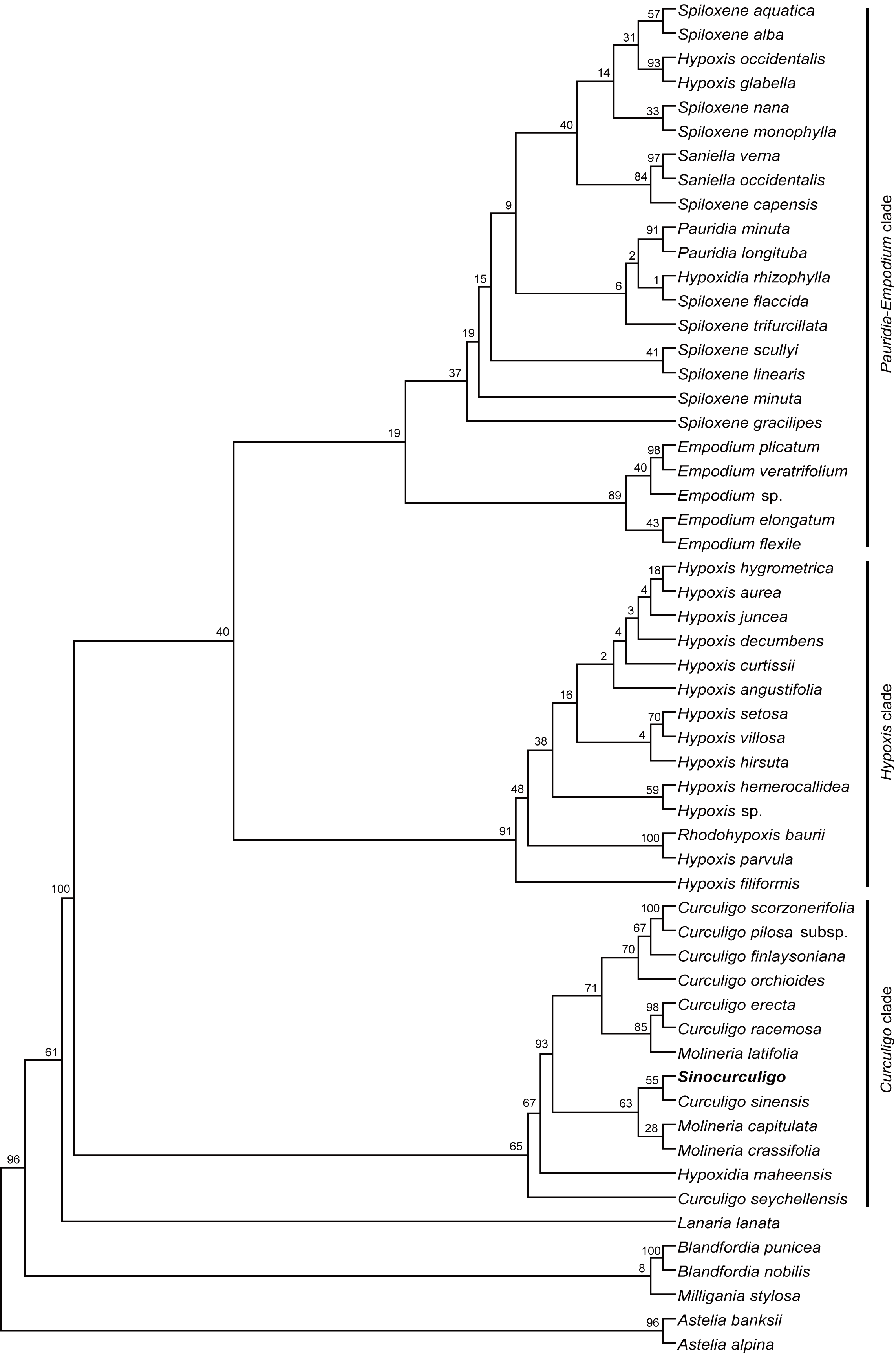

Supplement: Figure S17 — Maximum likelihood (ML) trees of trnL - F dataset of Hypoxidaceae, computed by RAxML with 100 bootstrap replicates. The bootstrap values are indicated above the branches. (TIF) [file pone.0038880.s017.tif]

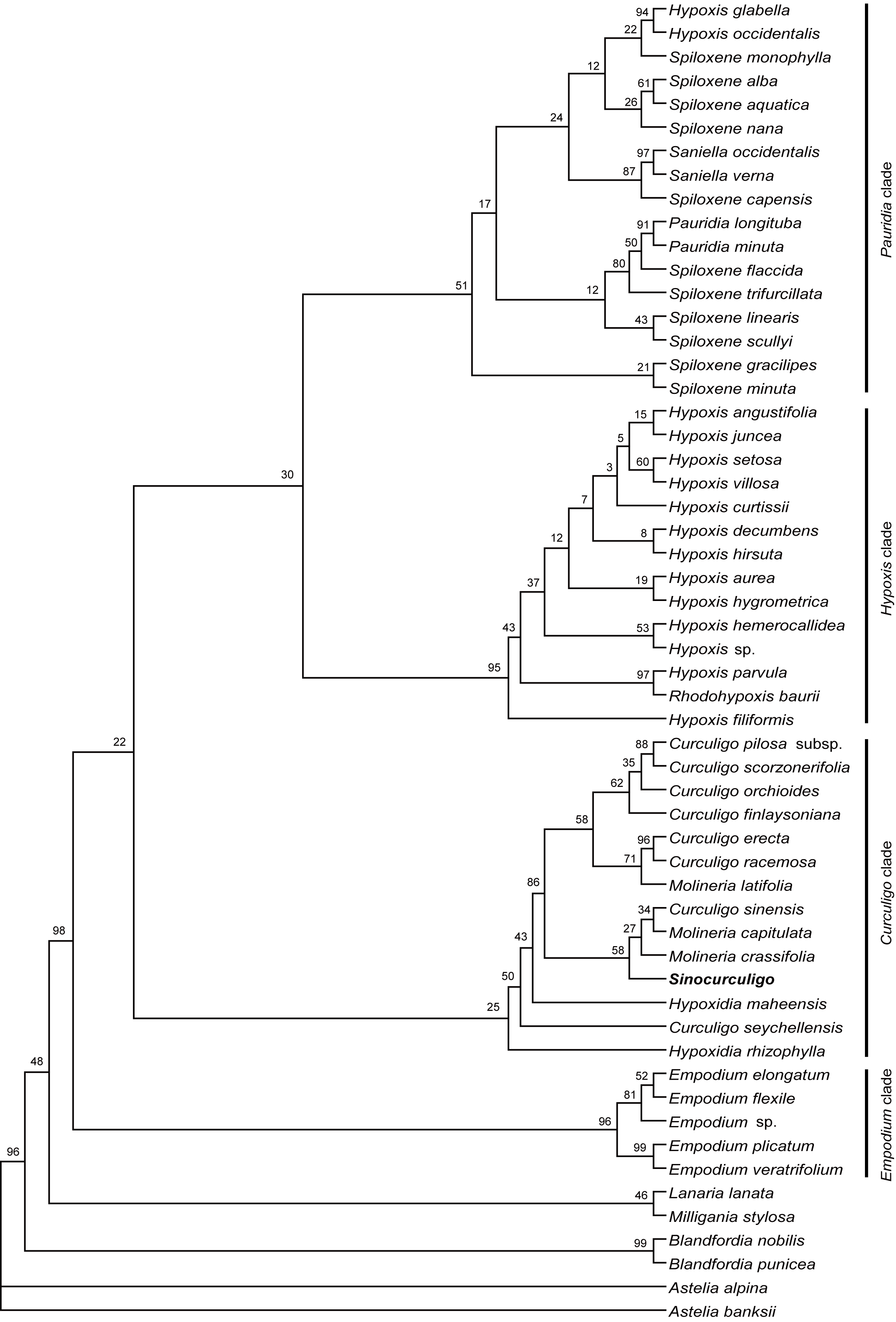

Supplement: Figure S18 — Strict consensus tree of the most parsimonious trees based on trnL - F dataset of Hypoxidaceae. The bootstrap values of the maximum parsimony analysis are indicated above the branches. (TIF) [file pone.0038880.s018.tif]

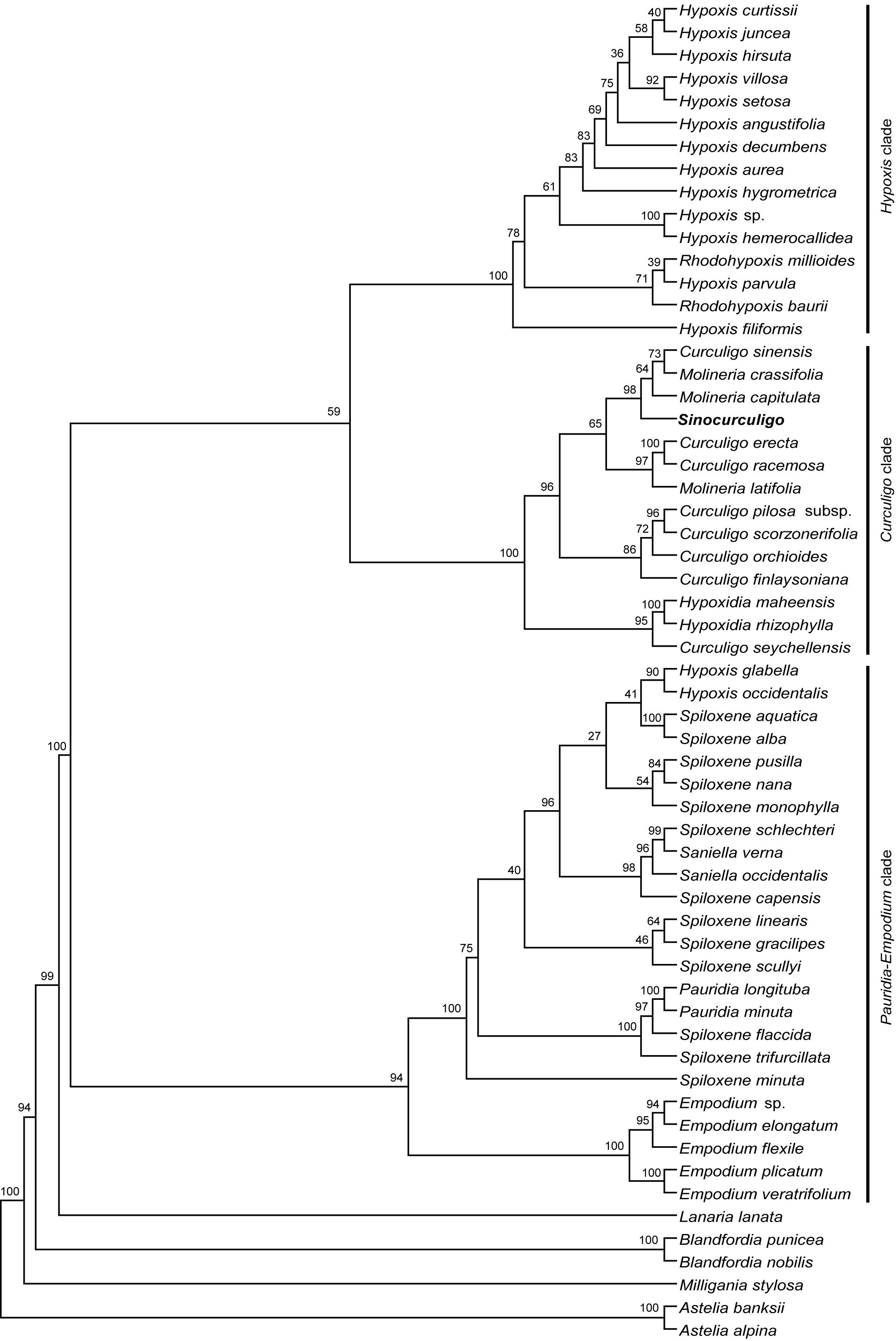

Supplement: Figure S19 — Maximum likelihood (ML) trees of combined dataset of Hypoxidaceae, computed by RAxML with 100 bootstrap replicates. Bootstrap values are indicated above the branches. (TIF) [file pone.0038880.s019.tif]

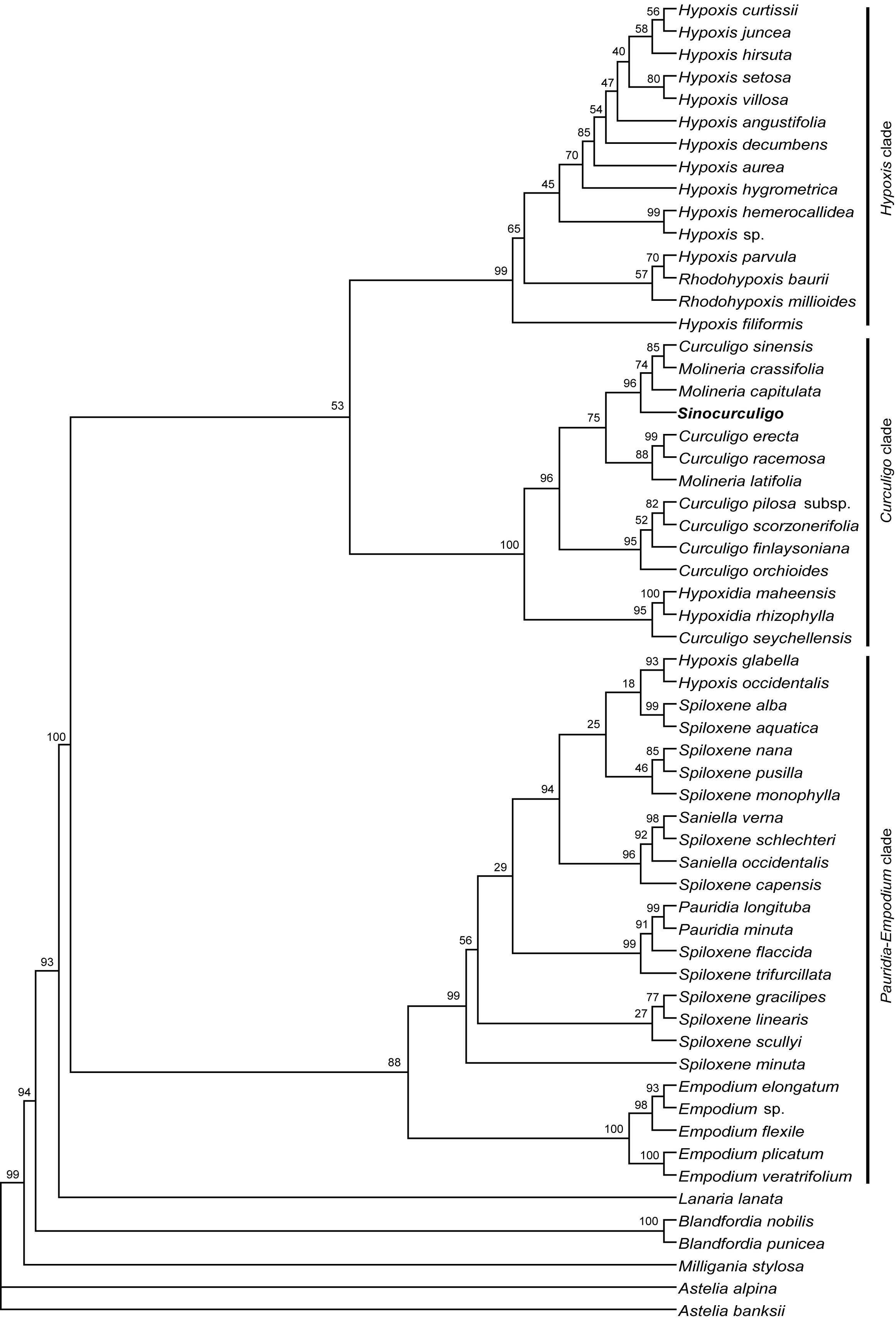

Supplement: Figure S20 — Strict consensus tree of the most parsimonious trees based on combined dataset of Hypoxidaceae. The bootstrap values of the maximum parsimony analysis are indicated above the branches. (TIF) [file pone.0038880.s020.tif]
