# Supplementary material for: Sinocurculigo, a New Genus of Hypoxidaceae from China Based on Molecular and Morphological Evidence
Source: PLoS One. 2012 Jun 27;7(6):e38880. doi: 10.1371/journal.pone.0038880 (PMC3384634; doi:10.1371/journal.pone.0038880)
Supplement: Table S1 — Species and gene regions sequenced for family-level analysis in this study. (DOC) [file pone.0038880.s021.doc]

**Table S1** Species and gene regions sequenced for family-level analysis in this study.

| genus | *matK* |  | | *rbcL* | |  |
| --- | --- | --- | --- | --- | --- | --- |
|  | Representative  species | Accession  number |  | | Representative species | Accession  number |
| *Silene* | *S. otites* | AY514848 | | *S. flos-cuculi* | | AY395549 |
| *Scleranthus* | *S. perennis* | AY514847 | | *S. annuus* | | AY270145 |
| *Illecebrum* | *I. verticillatum* | AY514849 | | *I. verticillatum* | | AY270143 |
| *Peperomia* | *P. pitcairnensis* | DQ212762 | | *P. pellucida* | | EF450306 |
| *Piper* | *P. pulchrum* | DQ882222 | | *P. chinense* | | EF450314 |
| *Macropiper* | *M. hooglandii* | DQ882228 | | *M. excelsum* | | AY298836 |
| *Arisaema* | *A. speciosum* | EU886502 | | *A. tortuosum* | | AF497109 |
| *Arum* | *A. rupicola* | EU886519 | | *A. pictum* | | EU193265 |
| *Lemna* | *L. trisulca* | AY034199 | | *L. gibba* | | AY034235 |
| *Typhonium* | *T. varians* | EU886575 | | *T. venosum* | | AF497113 |
| *Wolffia* | *W. elongata* | AY034220 | | *W. arrhiza* | | AY034254 |
| *Eminium* | *E. spiculatum* | EU886530 | | *E. spiculatum* | | AM905813 |
| *Agave* | *A. ghiesbreghtii* | HM640592 | | *A. ghiesbreghtii* | | HM640478 |
| *Chlorophytum* | *C. comosum* | AB029806 | | *C. suffruticosum* | | HM640493 |
| *Yucca* | *Y. filamentosa* | AY952431 | | *Y. schidigera* | | DQ069504 |
| *Agapanthus* | *A. africanus* | HM640599 | | *A. africanus* | | AY465699 |
| *Crinum* | *C. acaule* | EU527800 | | *Crinum asiaticum* | | HM640488 |
| *Galanthus* | *G. reginae-olgae* | FN663897 | | *Galanthus plicatus* | | Z69218 |
| *Lycoris* | *L. uydoensis* | HM640600 | | *L. squamigera* | | AF116971 |
| *Fritillaria* | *F. unibracteata* | GQ205149 | | *F. koidzumiana* | | AB034939 |
| *Calochortus* | *C. uniflorus* | AY624478 | | *C. minimus* | | Z77263 |
| *Gagea* | *G. gageoides* | EU912169 | | *G. lutea* | | AB034752 |
| *Erythronium* | *E. montanum* | AF485314 | | *E. japonicum* | | D28156 |
| *Lilium* | *L. speciosum* | AY624467 | | *L. superbum* | | AB034926 |
| *Tulipa* | *T. chrysantha* | AF485324 | | *T. turkestanica* | | AB037378 |
| *Babiana* | *B. unguiculata* | GQ381383 | | *B. virginea* | | GQ381626 |
| *Crocus* | *C. pulchellus* | AJ579941 | | *C. pulchellus* | | AJ309668 |
| *Gladiolus* | *G. caucasicus* | AY596622 | | *G. carneus* | | HM850029 |
| *Sisyrinchium* | *S. micranthum* | AJ579982 | | *S. micranthum* | | Z77290 |
| *Iris* | *I. tenax* | AY596647 | | *I. foetidissima* | | HM850074 |
| *Moraea* | *M. namaquamontana* | AJ579971 | | *M. saxicola* | | GQ285204 |
| *Ptychosperma* | *P. macarthurii* | AM114693 | | *P. burretianum* | | AY012495 |
| *Dypsis* | *D. lutescens* | AM114681 | | *D. lastelliana* | | AY012486 |
| *Calyptrocalyx* | *C. albertisianus* | AM114687 | | *C. stenocrista* | | AY012501 |
| *Nelumbo* | *N. nucifera* | AF543740 | | *N. nucifera* | | GQ997596 |
| *Curculigo* | *C. capitulata* | AY557207 | | *C. seychellensis* | | HM459546 |
| *Empodium* | *E. veratrifolium* | AY368374 | | *E. veratrifolium* | | HM459556 |
| *Hypoxis* | *H. hemerocallidea* | HM640657 | | *H. hemerocallidea* | | HM640539 |
| *Molineria* | *M. capitulata* | AB088783 | | *M. capitulata* | | HM640538 |
| *Pauridia* | – | – | | *P. minuta* | | HM639282 |
| *Rhodohypoxis* | *R. baurii* | HM640658 | | *R. baurii* | | HM640540 |
| *Sinocurculigo* | *S. taishanica* | JQ315817 | | *S. taishanica* | | JQ315819 |
| *Spiloxene* | – | – | | *S. scullyi* | | HM459579 |
| *Altensteinia* | *A. fimbriata* | EF065583 | | *A. fimbriata* | | FJ571315 |
| *Apostasia* | *A. wallichii* | AY557212 | | *A. wallichii* | | HM640552 |
| *Cymbidium* | *C. ensifolium* | AF263648 | | *C. ensifolium* | | AF074141 |
| *Cleistes* | *C. rosea* | AJ310006 | | *C. rosea* | | AF074128 |
| *Cypripedium* | *C. passerinum* | AF263649 | | *C. calceolus* | | EF370100 |
| *Goodyera* | *G. pubescens* | AJ543954 | | *G. repens* | | FJ571330 |
| *Habenaria* | *H. repens* | AJ310036 | | *H. repens* | | AF074177 |
| *Neuwiedia* | *N. veratrifolia* | AY557211 | | *N. veratrifolia* | | AF074200 |
| *Orchis* | *O. quadripunctata* | AY368385 | | *O. rotundifolia* | | AY149368 |
| *Paphiopedilum* | *P. haynaldianum* | AB176547 | | *P. glaucophyllum* | | AY557205 |
| *Paraholcoglossum* | *P. amesianum* | JN106350 | | *P. amesianum* | | HQ404488 |
| *Phragmipedium* | *P. ecuadorense* | AY918832 | | *P. ecuadorense* | | AY918856 |
| *Pleione* | *P. formosana* | AF302705 | | *P. formosana* | | F264173 |
| *Pogonia* | *P. ophioglossoides* | AJ310055 | | *P. ophioglossoides* | | EU498136 |
| *Pterostylis* | *P. nutans* | GQ405616 | | *P. nutans* | | AF074224 |
| *Vanilla* | *V. planifolia* | AJ310079 | | *V. planifolia* | | FN545561 |
| *Magnolia* | *M. villosa* | AB623296 | | *M. stellata* | | AF238057 |
