# Supplementary material for: Sinocurculigo, a New Genus of Hypoxidaceae from China Based on Molecular and Morphological Evidence
Source: PLoS One. 2012 Jun 27;7(6):e38880. doi: 10.1371/journal.pone.0038880 (PMC3384634; doi:10.1371/journal.pone.0038880)
Supplement: Table S2 — Species and gene regions sequenced for intra-Hypoxidaceae analysis and GenBank accession numbers. (DOC) [file pone.0038880.s022.doc]

**Table S2** Species and gene regions sequenced for intra-Hypoxidaceae analysis and GenBank accession numbers.

| Species | *rbcL* | *trnL-F* | *trnS-G* |
| --- | --- | --- | --- |
| Hypoxidaceae |  |  |  |
| *Curculigo erecta* | HM459539 | HM459485 | HM459437 |
| *Curculigo ﬁnlaysoniana* | HM459540 | HM459486 | – |
| *Curculigo orchioides* | HM459541 | HM459487 | HM459438 |
| *Curculigo pilosa* ssp*. major* | HM459542 | HM459488 | HM459439 |
| *Curculigo racemosa* | HM459543 | HM459489 | HM459440 |
| *Curculigo scorzonerifolia* | HM459544 | HM459490 | HM459441 |
| *Curculigo seychellensis* | HM459545 | HM459491 | HM459442 |
| *Curculigo sinensis* | HM459547 | HM459493 | HM459444 |
| *Empodium elongatum* | HM459553 | HM459495 | HM459445 |
| *Empodium ﬂexile* | HM459554 | HM459494 | HM459446 |
| *Empodium plicatum* | HM459555 | HM459496 | HM459447 |
| *Empodium veratrifolium* | HM459556 | HM459497 | HM459448 |
| *Empodium* sp. | HM459557 | HM459498 | HM459449 |
| *Hypoxidia maheensis* | HM459558 | HM459499 | HM459450 |
| *Hypoxidia rhizophylla* | HM459559 | HM459500 | HM459451 |
| *Hypoxis angustifolia* | HM459560 | HM459501 | HM459452 |
| *Hypoxis aurea* | HM459562 | HM459503 | HM459454 |
| *Hypoxis curtissii* | Z73702 | HM459504 | HM459455 |
| *Hypoxis decumbens* | HM459563 | HM459505 | HM459456 |
| *Hypoxis ﬁliformis* | HM459561 | HM459502 | HM459453 |
| *Hypoxis glabella* | Y14989 | HM459506 | HM459457 |
| *Hypoxis hemerocallidea* | HM459564 | HM459507 | HM459458 |
| *Hypoxis hirsuta* | HM459565 | HM459508 | HM459459 |
| *Hypoxis hygrometrica* | HM459566 | HM459509 | HM459460 |
| *Hypoxis juncea* | HM459567 | HM459510 | HM459461 |
| *Hypoxis occidentalis* | HM459568 | HM459511 | HM459462 |
| *Hypoxis parvula* | HM639281 | HM570029 | HM639292 |
| *Hypoxis setosa* | HM459569 | HM459512 | HM459463 |
| *Hypoxis villosa* | HM459570 | HM459513 | HM459464 |
| *Hypoxis* sp. | HM459571 | HM459514 | HM459465 |
| *Molineria capitulata* | Z73701 | HM459515 | HM459466 |
| *Molineria crassifolia* | HM459548 | HM459516 | HM459467 |
| *Molineria latifolia* | HM459550 | HM459517 | HM459468 |
| *Molineria latifolia* | HM459549 | HM459518 | – |
| *Pauridia longituba* | HM459572 | HM459521 | HM459469 |
| *Pauridia minuta* | HM639282 | HM570030 | HM639293 |
| *Rhodohypoxis baurii* | HM459573 | HM459523 | HM459471 |
| *Sinocurcurligo taishanic* | JQ315819 | JQ315820 | JQ315818 |
| *Saniella occidentalis* | HM639284 | HM639301 | HM639294 |
| *Saniella verna* | HM459574 | HM459524 | HM459472 |
| *Spiloxene alba* | HM459575 | HM459525 | HM459473 |
| *Spiloxene aquatica* | HM639285 | HM639302 | HM639295 |
| *Spiloxene capensis* | HM459576 | HM459526 | HM459474 |
| *Spiloxene ﬂaccida* | HM459577 | HM459527 | HM459475 |
| *Spiloxene gracilipes* | HM639286 | HM570032 | HM639296 |
| *Spiloxene linearis* | HM639287 | HM639303 | HM639297 |
| *Spiloxene minuta* | [HM639288](http://www.sciweb.nybg.org/science2/IndexHerbariorum.asp) | HM570033 | HM639298 |
| *Spiloxene monophylla* | [HM459578](http://www.sciweb.nybg.org/science2/IndexHerbariorum.asp) | HM459528 | HM459476 |
| *Spiloxene nana* | [HM639289](http://www.sciweb.nybg.org/science2/IndexHerbariorum.asp) | HM570034 | HM639299 |
| *Spiloxene pusilla* | [HM639290](http://www.sciweb.nybg.org/science2/IndexHerbariorum.asp) | – | – |
| *Spiloxene schlechteri* | [HM639291](http://www.sciweb.nybg.org/science2/IndexHerbariorum.asp) | – | HM459477 |
| *Spiloxene scullyi* | [HM459579](http://www.sciweb.nybg.org/science2/IndexHerbariorum.asp) | HM459529 | HM459478 |
| *Spiloxene trifurcillata* | [HM570028](http://www.sciweb.nybg.org/science2/IndexHerbariorum.asp) | HM639304 | HM639300 |
| Outgroups |  |  |  |
| Asteliaceae |  |  |  |
| *Astelia alpina* | HM459580 | HM459530 | HM459479 |
| *Astelia banksii* | [Y14983](http://www.sciweb.nybg.org/science2/IndexHerbariorum.asp) | HM459531 | HM459480 |
| *Milligania stylosa* | Z73693 | HM459533 | HM459481 |
| Blandfordiaceae |  |  |  |
| *Blandfordia nobilis* | Y14984 | HM459534 | HM459482 |
| *Blandfordia punicea* | Z73694 | HM459535 | HM459483 |
| Lanariaceae |  |  |  |
| *Lanaria lanata* | HM459581 | HM459536 | HM459484 |
